# *De novo* Transcriptome Assembly of *Phomopsis liquidambari* Provides Insights into Genes Associated with Different Lifestyles in Rice (*Oryza sativa* L.)

**DOI:** 10.3389/fpls.2017.00121

**Published:** 2017-02-06

**Authors:** Jun Zhou, Xin Li, Yan Chen, Chuan-Chao Dai

**Affiliations:** Jiangsu Key Laboratory for Microbes and Functional Genomics, Jiangsu Engineering and Technology Research Center for Industrialization of Microbial Resources, College of Life Sciences, Nanjing Normal UniversityNanjing, China

**Keywords:** *Phomopsis liquidambari*, rice, endophyte, saprophyte, transcriptome, ecological adaptation

## Abstract

The mechanisms that trigger the switch from endophytic fungi to saprophytic fungi are largely unexplored. Broad host range *Phomopsis liquidambari* is established in endophytic and saprophytic systems with rice (*Oryza sativa* L.). Endophytic *P. liquidambari* promotes rice growth, increasing rice yield and improving the efficiency of nitrogen fertilizer. This species's saprophytic counterpart can decompose rice litterfall, promoting litter organic matter cycling and the release of nutrients and improving the soil microbial environment. Fluorescence microscopy, confocal laser scanning microscopy and quantitative PCR investigated the colonization dynamics and biomass of *P. liquidambari* in rice *in vivo*. *P. liquidambari* formed infection structures similar to phytopathogens with infected vascular tissues that systematically spread to acrial parts. However, different from pathogenic infection, *P. liquidambari* colonization exhibits space restriction and quantity restriction. Direct comparison of a fungal transcriptome under three different habitats provided a better understanding of lifestyle conversion during plant-fungi interactions. The isolated total RNA of Ck (pure culture), EP (endophytic culture) and FP (saprophytic culture) was subjected to Illumina transcriptome sequencing. To the best of our knowledge, this study is the first to investigate *Phomopsis* sp. using RNA-seq technology to obtain whole transcriptome information. A total of 27,401,258 raw reads were generated and 22,700 unigenes were annotated. Functional annotation indicated that carbohydrate metabolism and biosynthesis of secondary metabolites played important roles. There were 2522 differentially expressed genes (DEGs) between the saprophytic and endophytic lifestyles. Quantitative PCR analysis validated the DEGs of RNA-seq. Analysis of DEGs between saprophytic and endophytic lifestyles revealed that most genes from amino acids metabolism, carbohydrate metabolism, fatty acid biosynthesis, secondary metabolism and terpenoid and steroid biosynthesis were up-regulated in EP. Secondary metabolites of these pathways may affect fungal growth and development and contribute to signaling communication with the host. Most pathways of xenobiotic biodegradation and metabolism were upregulated in FP. Cytochrome P450s play diverse vital roles in endophytism and saprophytism, as their highly specialized functions are evolutionarily adapted to various ecological niches. These results help to characterize the relationship between fungi and plants, the diversity of fungi for ecological adaptations and the application prospects for fungi in sustainable agriculture.

## Introduction

In farmland ecosystems, intensive agriculture weakens the self-cycle capacity of soil nutrients, forcing farmers to devote effort to collecting and burning plant litter and applying fertilizer to farmland. This leads to the waste of natural organic resources, atmospheric and environmental pollution and soil quality deterioration, which are unfavorable for sustainable agriculture. Therefore, it is necessary to find an appropriate medium to decompose plant residues in farmland in order to increase soil available nutrients.

Endophytic fungi and saprophytic fungi usually play important ecological functions in living plant tissue and dead plant material. Many studies have investigated the relationship between endophytes and saprophytes and have hypothesized that endophytes become saprophytic after senescence of host tissues (Hyde et al., 2007). This may be due to modification of host tissues during senescence, allowing fungal hyphae to penetrate the epidermis and colonize the surface of the host. Promputtha et al. (2007) isolated common endophytes from *Magnolia liliifera, Phomopsis, Guignardia, Fusarium*, and *Colletotrichum* that have a high degree of sequence similarity and are phylogenetically relevant to the corresponding saprophyte. These results suggest that some endophytes might alter their ecological strategies and adopt a saprophytic lifestyle. Promputtha et al. (2010) also reported that nine endophytes, *Phomopsis* sp. 2, *Phomopsis* sp. 6, *Phomopsis* sp. 10, *Guignardia mangiferae, Corynespora cassiicola, Leptosphaeria* sp., *Fusarium* sp. 1, *Colletotrichum gloeosporioides* and *Colletotrichum* sp. 2 were morphologically similar and phylogenetically related to saprophytes. These endophytes and their saprobic counterparts produce the same degrading enzymes and a similar isoform of β-mannanase. Fungal succession is relevant to enzyme production patterns during leaf decomposition, and the occurrence of saprophytes is related to enzyme production from endophytes. This provides further convincing evidence that endophytes can change their lifestyle to become saprophytes.

Lipids are an important component of all living cells that offer a structural basis for cell membranes and fuel for metabolism and have a role in cell signaling. Membrane lipid synthesis is a prerequisite of symbiosis, and the performance of the membrane depends on lipid composition (Wewer et al., 2014). Fatty acids and modified fatty acids are important molecules for pathogen colonizing plants whose functions include signaling, energy sources and virulence factors (Uranga et al., 2016). The oxylipins are a vast diversified family of secondary metabolites derived from oxidation of unsaturated fatty acids or further conversion (Tsitsigiannis and Keller, 2007). In fungi, precursors of oxylipins are usually linoleic acid, oleic acid and α-linolenic acid (Pohl and Kock, 2014). Fungal oxylipins can be used as secondary metabolites that participate in infection processes, biotrophy and necrotrophy (Oliw et al., 2016). Fungi produce a series of secondary metabolites and small molecules that may not be directly required for growth, but play important roles in signal transduction, development and organism interaction. The cytochrome P450 enzyme system is thought to play various functions in biosynthesis of secondary metabolites and participated in biodegradation of lignin and various xenobiotics (Martinez et al., 2009).

Though most endophytes depend on readily available compounds such as soluble sugar to grow, xylariaceous endophytes can degrade cellulose and lignin (Promputtha et al., 2010). Hence, endophytes that produce enzymes to decompose lignin and cellulose could decompose host tissue and persist as saprophytes following host senescence. Our research shows that the endophyte *Phomopsis liquidambari* B3 can establish a symbiotic relationship with rice (*Oryza sativa* L.), systematically colonizing roots and aerial parts (Yang et al., 2014b), which promotes the growth of rice, increasing yield and significantly reducing application of nitrogen fertilizer (Yang et al., 2014a, 2015; Siddikee et al., 2016). In the saprophytic phase, the fungus can decompose rice straw, promote litter organic matter cycling and the release of nutrients, improve soil microbial environments (Chen et al., 2013a) and secrete laccase, cellulase and polyphenol oxidase. Dai et al. (2010a) investigated the capability to form cavities on the straw surface and the condition for laccase production in *P. liquidambari*, suggested that endophytes can form a series of cavities on straw to decompose plant materials by producing laccase. In addition, *P. liquidambari* also secretes degradative enzymes for phenanthrene (Dai et al., 2010b), indole (Chen et al., 2013c; Wang et al., 2014), ferulic acid (Xie and Dai, 2015), 4-hydroxybenzoic acid (Chen et al., 2011) and phytoestrogen luteolin (Wang et al., 2015) among others. The fungus utilizes these compounds as the sole carbon source for growth. Chen et al. (2013c) shows that the degradation rate of indole in endophyte B3 over 120 h in a pure culture condition was 41.7%. The exogenous addition of plant litter significantly increased the ratio of indole degradation within 60 h to 99.1%, indicating the utility of litter-induced fungi to produce laccase and lignin peroxidase to non-specifically decompose nitrogen heterocyclic compounds.

However, comparisons of different types of plant-fungal interactions in the same plant species are limited because saprophytic systems and mutualistic systems are separated in various plants. Therefore, it will be valuable to perform experiments studying saprophytism and mutualism in a single plant species to directly compare endophytic and saprophytic plant-fungi interactions. Few studies have directly compared two different plant-microbe interactions in a single plant species. Furthermore, because only a small part of plant cells are colonized and it is difficult to accurately detect expression levels of fungal genes in colonized tissue, the detection of gene expression profiles and elucidation of interactional mechanisms during the endophytic lifestyle transition at different stages in associated host tissues remain poorly understood. Recently, “omics” approaches have been used to better understand endophyte-plant interactions (Kaul et al., 2016).

Rice is a representative gramineous plant, the staple food for approximately half the global population and a model material in agricultural microbiology research. *P. liquidambari* is a broad-spectrum endophyte that is typically used to study the switch from endophytism to saprophytism. We have established experimental systems to study the endophytic and saprophytic interaction of *P. liquidambari* B3 with a rice single host. Colonization dynamics and distribution in rice *in vivo* were monitored by green fluorescent protein (GFP)-tagged *P. liquidambari*. To discuss the differences in gene expression in *P. liquidambari* B3 interactions with rice under the two conditions of endophytism and saprophytism transcriptome sequencing technology and digital gene expression profiles were used. Endophytes are known to establish a symbiosis with the host through a series of regulatory mechanisms, as there is a sizable difference in performance between mutualistic fungi in the host and the common saprophyte. However, there is insufficient research into the switch from endophyte to saprophyte function in senescent plant litter. Clarification of this scientific problem has great significance for understanding the relationship between endophytes and plants and for documenting the diversity of endophytes.

## Materials and methods

### Fungal strain and transformation

*P. liquidambari* B3 was isolated from the inner bark of *Bischofia polycarpa*. It was stored on a potato dextrose agar slant (200 g potato extract, 20 g glucose, 20 g ager per liter, pH 7.0) at 4°C. The fungus was activated in potato dextrose broth (200 g potato extract, 20 g glucose per liter, pH 7.0) and cultured for 48 h at 28°C with a rotation speed of 180 rpm.

The transformation vector plasmid for filamentous fungi pCT74 expresses sGFP under the control of the *ToxA* promoter. It contains the hygromycin B resistance gene *hph*, which encodes an aminoglycosidic antibiotic and is derived from *Streptomyces hygroscopicus*. This gene has been used for selection and maintenance of transformed prokaryotic and eukaryotic cells. Protoplast preparation and transformation were performed as described by Yang et al. (2014b) with some modifications.

### Inoculation, co-culture and microscopy

The rice cultivar used in this study was “Wuyunjing 21”. Rice seeds were dehusked and surface-sterilized in 75% ethanol for 15 min, bleached in a 6% sodium hypochlorite solution (6% available chlorine) for 10 min, rinsed repeatedly in sterile distilled water, and planted in 1/2 Murashige & Skoog (MS) 0.7% agarose medium supplemented with 30 mM sucrose for 4 days. The seedlings were kept vertical at 25°C under a 16 h of light at 22°C and 8 h of dark. Seedlings of roughly the same size were transferred to 1/2 MS in a square petri dish (13 cm in width, 13 cm in length). Each plate of five plants inoculated with 7-mm GFP-B3 mycelial disks were placed near the plant roots on the medium. Potato dextrose agar disks of equal size were used as a control. All treatments were replicated five times. Rice shoots and roots were sampled and processed for microscopy.

An Axio Imager A1 fluorescence microscope (Zeiss, Jena, Germany) was used for observing the fungal structures as described previously by Yang et al. (2014b). Confocal laser scanning microscopy was performed using a Ti-E microscope with an A1 confocal system (Nikon, Tokyo, Japan) to monitor the infection process. GFP and FITC images of rice shoots and roots were captured simultaneously using 488 nm excitation with an argon laser and fluorescence detection at 543.5 nm. Images were processed using Adobe Photoshop CS6 (Adobe, San Jose, CA, USA).

### Quantification of *P. liquidambari* biomass in rice roots and shoots by quantitative PCR

*P. liquidambari*-infected roots and shoots were harvested at 0, 3, 7, 14, 21, and 28 dai (days after inoculation). The biomass of *P. liquidambari* in the infected plant tissue was quantified using quantitative PCR (qPCR) according to Yang et al. (2014b). DNA was extracted after grinding tissue powder with a Multisource Genomic DNA Miniprep Kit (Axygen). A primer set suitable for qPCR was designed based on a *P. liquidambari*-specific ITS locus (Bf1 and Br1) (Table S1). PCR amplified products were cloned into the pMD® 19-T vector (Takara, Otsu, Japan) and expressed in competent DH5αcells. Positive clones were screened and plasmids were extracted using a SanPrep Column Plasmid Mini-Prep Kit. A dilution range of the plasmids from 1.3 × 10^2^ to 1.3 × 10^8^ copies was used to make a standard curve. In the qPCR reaction system (20 μL), gDNA was mixed with SYBR® Green Master Mix (Vazyme, Nanjing, China), primers, and ddH_2_O. The PCR procedure was as follows: 94°C (1 min) for one cycle; 94°C (15 s), 60°C (45 s), and 72°C (30 s) for 45 cycles; melting curve analysis from 72 to 60°C in 0.5°C decrements. The amplification of a single PCR product was validated using 1.5% gel electrophoresis.

### Endophytic and saprophytic systems of *P. liquidambari* interact with rice

Activated *P. liquidambari* was filtered with 8-layer gauze. Fungal mycelia were cleaned three times with sterile deionized water and 0.10 g mycelia (0.01 g dry biomass) were weighed and used for callus inoculation. In addition, 0.30 g mycelia (0.03 g dry biomass) were weighed and inoculated in 20 mL sterile water as a fungus seed solution for litterfall inoculation; 0.30 g mycelia were weighed and inoculated in 1 × NB solid medium for 3 days and regarded as a control treatment (Ck).

The endophytic lifestyle was studied using tissues cultures of host rice in dual culture *in vitro* based on previous research (Sieber et al., 1990; Peters and Schulz, 1998; Nawrot-Chorabik et al., 2016). Inoculated rice seeds at the surface were sterilized in callus solid medium (1 × NB solid medium, 2 mg L^−1^ 2,4-D, 30 g L^−1^ sucrose, 10 g L^−1^ agar, pH 7) and cultivated for 20 days at 28°C. The callus was stripped from rice seeds at germination and a yellow soft callus appeared at the base of medium. This was placed on new callus solid medium and cultivated for 50 days under 28°C. When a callus formed (Figure S1), *P. liquidambari* mycelium was inoculated on top of the callus with tweezers and cultivated for 3 days at 28°C. At this stage, the fungus was checked to ensure that it grew on the surface of the callus but did not contact the medium. The mycelium was stripped from the surface of the callus with delicate tweezers, and this sample was used as the fungal mycelium of the *P. liquidambari* callus culture (EP).

The saprophytic system was derived using the culture fungus method of Chen et al. (2013a) with litter. Collected rice litterfall completely withered from the ground of the rice experimental plot. The moisture content of rice litterfall was 7.6%. The litterfall surface was washed with sterile deionized water and cut into 1 cm × 1 mm segments. Weighed 0.5-g litterfall samples were added to a 250-mL triangular flask with 100 mL 1 × NB liquid medium (pH 5.5) and sterilized for 20 min at 121°C. A 2-mL sample of *P. liquidambari* was inoculated into the seed solution and cultivated for 3 days at 28°C and 160 rpm. The mycelium pellet was removed from the liquid medium with tweezers and washed clean. This sample was used as a litterfall-cultivated fungal mycelium of *P. liquidambari* (FP).

### RNA extraction

Total RNA was isolated from Ck, EP and FP using a Fungal RNA extraction kit (E.Z.N.A. Total RNA Kit I, OMEGA, USA) and treated with DNase I. The quality and concentration of extracted RNA were examined using agarose gel electrophoresis and a spectrophotometer (OneDrop™ OD-2000+, China), and eligible groups were used for Illumina sequencing.

### cDNA library construction and sequencing

The mRNA of the total RNA was purified using magnetic Oligo (dT) beads. This mRNA was mixed with the fragmentation buffer, the mRNA was fragmented into short fragments. The cDNA was synthesized using mRNA fragments as templates. Short fragments were purified and resolved with EB buffer for sticky-end preparation and single nucleotide A addition. Subsequently, the short fragments were connected with adapters, and suitable fragments were selected as templates for PCR amplification. Quantification and qualification of the sample library was performed using an ABI StepOnePlus Real-Time PCR System and an Agilent 2100 Bioanalyzer. The library was sequenced using Illumina HiSeq™ 2000.

### Sequence annotation

Image data output from Illumina sequencing was transformed by base calling into raw reads. Clean reads were obtained by removing dirty reads that contained adapters or unknown or low quality bases. Transcriptome *de novo* assembly was carried out with Trinty (Grabherr et al., 2011) and a k-mer library was constructed. The highest frequency k-mer was selected to assemble contigs and then mapped with clean reads. Paired-end reads were used to fill gaps in the scaffolds to assemble contigs to unigenes. Non-redundant unigenes were acquired by further processing of sequence splicing and removal of redundancy. All-unigenes were generated after gene family clustering. Unigene sequences were aligned with blastx (*e* < 0.00001) to protein databases including non-redundant databases (NR), Swiss-Prot, the Kyoto Encyclopedia of Genes and Genomes (KEGG) and the Clusters of Orthologous Groups of proteins (COG), and aligned by blastn (*e* < 0.00001) to the nucleotide databases nt. Gene Ontology (GO) functional annotation was achieved using NR annotation by Blast2GO (Conesa et al., 2005). GO functional classification was achieved using WEGO software (Ye et al., 2006).

### Identification of differentially expressed genes

The FPKM method was used to calculating unigene expression (Mortazavi et al., 2008). An algorithm to identify differentially expressed genes (DEGs) between the two samples was used according to the method of Audic and Claverie (1997). In our analysis, the genes with false discovery rates (FDR) ≤ 0.001 and ratios larger than 2 were regarded as significant DEGs. We mapped all DEGs to terms in the GO database and KEGG database for enrichment analysis.

### Quantitative real-time PCR analysis

To validate the DEGs obtained by Solexa RNA-seq, 20 genes (Table S1) were subjected to quantitative real-time PCR analysis using an ABI PRISM 7500 Real-time PCR System (Applied Biosystems). *P. liquidambari* β-actin (Table S1) was used as the endogenous control. cDNA synthesis was carried out using the same RNA samples as those used for digital gene expression profiling (DGE) experiments. The corresponding primers were designed using Primer Premier 6.0 and listed in Table S1. The reaction mixture (20 μl) contained 10 μl of SYBR® Green Master Mix (Vazyme, Nanjing, China), 0.4 μM of forward and reverse primers, and 0.2 μl of cDNA template. The amplification programs were performed with the following parameters: 95°C for 30 s; 95°C for 5 s, 60°C for 40 s (40 cycles) and followed by melting curve analysis from 60 to 95°C in 0.5°C increments. Each reaction was run in triplicate, including a negative control. The relative expression levels of genes were calculated using the 2^−ΔΔCT^ method.

## Results

### Infection and colonization process of rice plants systemically by *P. liquidambari*

**Figure 1:**
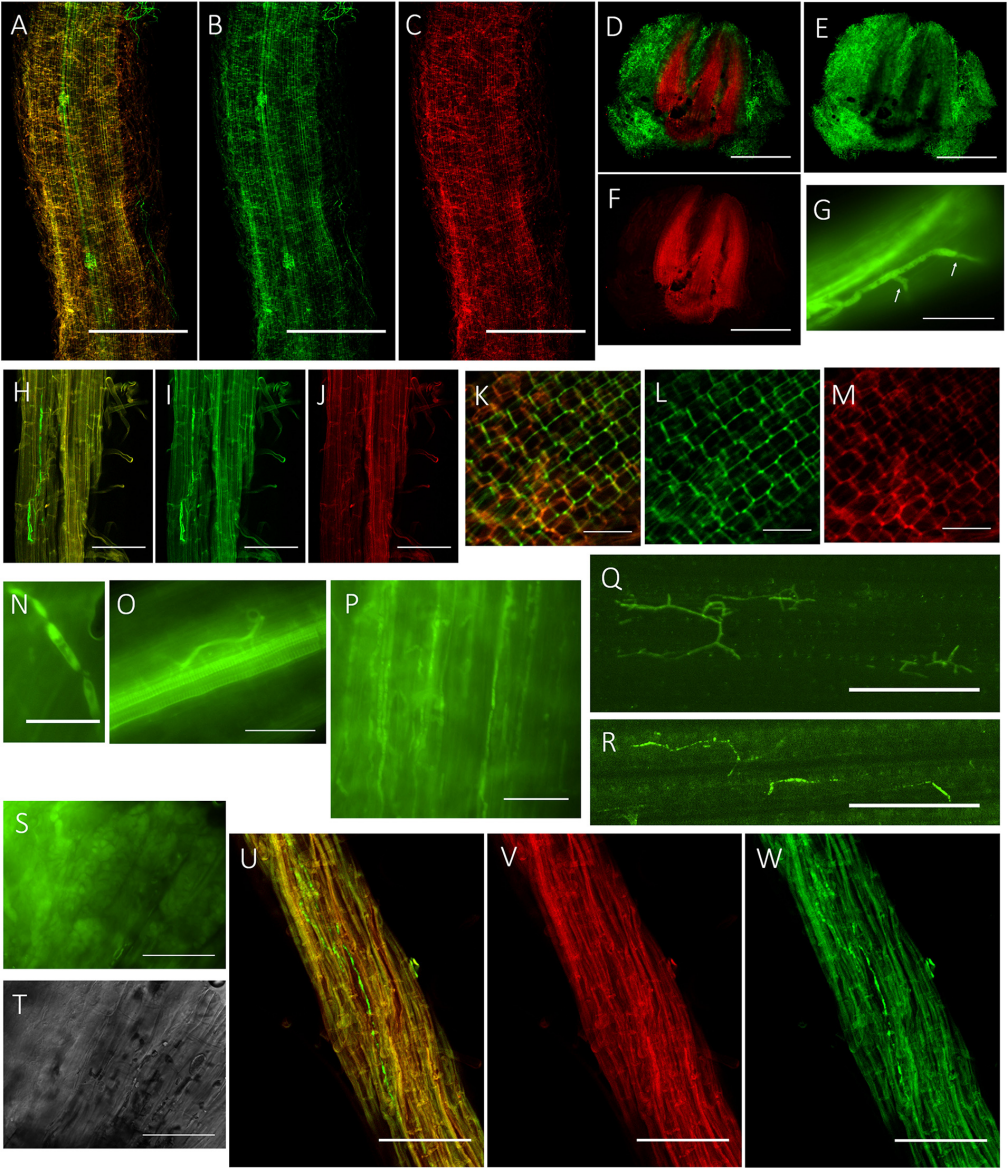
**Colonization pattern of ***P. liquidambari*** in rice roots and shoots. (A–C)** Distribution of GFP- *P. liquidambari* hyphae on the root surface and infection of root-hair; 1–3 dai. **(A)** Overlay channel. **(B)** Green fluorescence channel. **(C)** Red fluorescence channel. Bar, 100 μm. **(D–F)** Cross-section of root tip, runnner hypha interweaved to form hyphal network. **(D)** Overlay channel. **(E)** Green fluorescence channel. **(F)** Red fluorescence channel. Bars, 100 μm. **(G)** Penetration peg (arrow). Bar, 25 μm. **(H–J)** Intracellular and intercellular growth of hypha along the direction parallel to the root spindle spread from one cell to another; 4–15 dai. Bar, 25 μm. **(H)** Overlay channel. **(I)** Green fluorescence channel. **(J)** Red fluorescence channel. **(K–M)** Hyphae branching in the intercellular space. **(K)** Overlay channel. **(L)** Green fluorescence channel. **(M)** Red fluorescence channel. Bar, 50 μm. **(N)** Neck-like constriction, Bar, 10 μm. **(O)** Penetration of the center of the root spindle. Bar, 25 μm. **(P–R)** Colonization of hyphae in acrial parts. **(P)** Colonization of hyphae in stem cells. **(Q,R)** Colonization of hyphae in leaf cells. Bar, 50 μm. **(S,T)** Fluorescence microscopy of root cells were crowded with mycelium and sclerotium (>15 dai). **(S)** DIC channel. **(T)** bright field channel. Bar, 25 μm. **(U,V)** Colonization of hyphae in senescence rice root (>50 dai). **(U)** Overlay channel. **(V)** Red fluorescence channel. **(W)** Green fluorescence channel. Bar, 50 μm.

To visualize the infection process of *P. liquidambari in planta*, we first obtained transgenic fungal strains by constitutively expressing cytoplasmic GFP (GFP-B3). In the early stage of infection (1–3 dai), *P. liquidambari* hyphae were only distributed on the root surface, especially the root-hair zone, infected root-hairs and infected epidermis (Figures 1A–C). A large number of runner hypha interweaved together to form a hyphal network (Figures 1D–F and Video S1). Runner hypha were distributed in the low-lying area between the cells of root epidermal layers, growing along the longitudinal axis direction of the root and invading cells using a penetration peg (Figure 1G). In the middle stage of infection (4–15 dai), hyphae beginning intracellular and intercellular infection spread from epidermal layers to the cortex and finally to the endodermis (Figures 1H–J). In the epidermis and cortex, hyphae can undergo intracellular and intercellular growth along the direction parallel to the root spindle spread from one cell to another, branching in the intercellular space, and then continue to grow (Figures 1K–M). When it penetrated the cell wall, hyphae appeared with neck-like constrictions (Figure 1N). Parts of strong hypha entered the center of the root spindle and then penetrated the vascular bundle into the acrial part (Figures 1O–R). In late-stage colonization (>15 dai), which is associated with programmed cell death, the vast majority of epidermal cells and parts of outer cortex cells were crowded with mycelium and sclerotium (Figures 1S,T). Colonization of *P. liquidambari* was still observed in the senescence root (>50 dai) (Figures 1U–W).

### Quantification of *P. liquidambari* biomass in rice tissues

**Figure 2:**
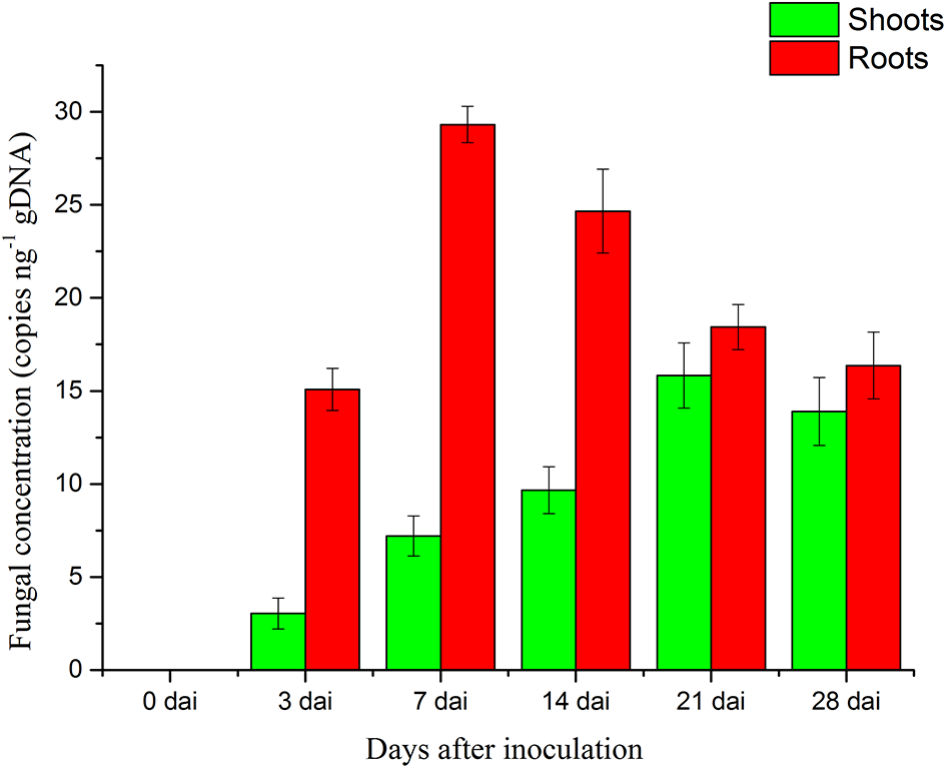
**Concentration of ***P. liquidambari*** in infected rice tissue at various time points (0, 3, 7, 14, 21, 28 dai)**. Values are means ± SD from three biological replicates.

The concentration of *P. liquidambari* within plantlets is expressed as the number of *P. liquidambari*-specific ITS copies per ng total (plantlet + fungal) genomic DNA in the qPCR analysis. The concentration of endophytes in roots was always higher than in shoots from 0 to 28 dai. In roots, a significant increase from 0 to 7 dai was followed by a moderate decrease. A a moderate increase in shoots occurred from 0 to 21 dai and then reached a steady state (Figure 2).

### Illumina RNA-sequencing and read assembly

**Figure 3:**
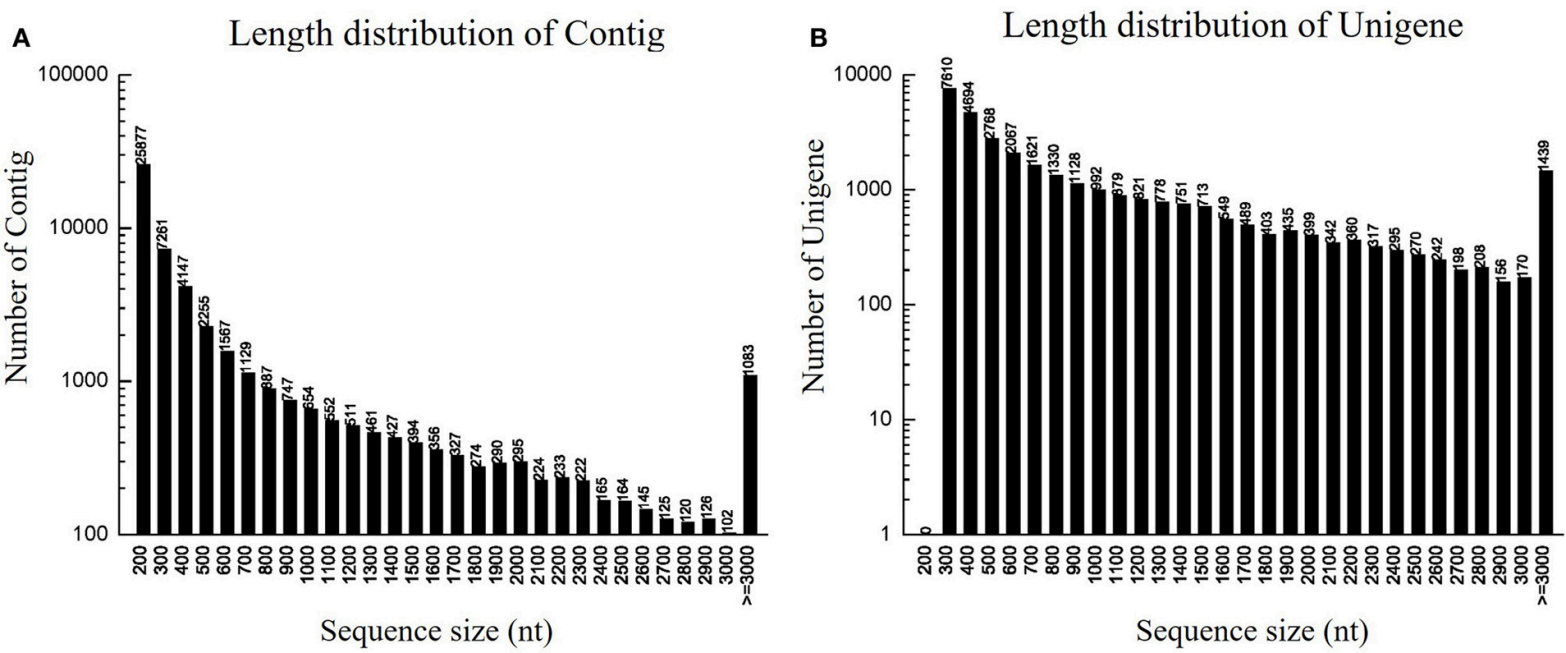
**Length distribution of contigs and unigenes. (A)** Length distribution of contigs. Horizontal coordinate is contig length and vertical coordinate is contig number. **(B)** Length distribution of unigenes. Horizontal coordinate is unigene length and vertical coordinate is number of unigenes.

To identify DEGs related to lifestyle, we mixed total RNA extracted from Ck, FP and EP of *P. liquidambari* hyphae equally for transcriptome sequencing. A total of 27,401,258 raw reads were generated. After filtering, 26,109,074 clean reads were obtained, for a total of 2,349,816,660 bp clean nucleotides. The Q20 percentage was 96.47% and the GC percentage was 56.45%. After editing and quality checking, 26 million 90 bp clean reads were assembled into 51,120 contigs with a mean length of 487 bp. The N50 of contigs was 1240 bp, where larger numbers are better for the quality of assembly. Using paired-end joining and gap filling, the contigs were further assembled into 32,424 unigenes with a mean length of 945 bp, including 7946 distinct clusters and 24,478 distinct singletons. The N50 of unigenes was 1574 bp, indicating that the assembly results were desirable (Table 1). The assembled sequence length is one evaluative criteria of assembly quality. The size distribution of the contigs and unigenes are shown in Figure 3. By comparing the length distribution proportion of contigs and unigenes, we found that contigs from 100 to 200 bp accounted for 50.62%, greater than 500 bp that accounted for 22.65% (Table S2). However, the length of unigenes obtained from further assembly, of which of 100–500 bp accounted for just 46.48%, were all greater than 200 bp and the proportion greater than 1000 bp was over 31.51% (Table S3), indicating that the assembly quality of unigenes that assembled from contigs was high.

**Table 1:** **Statistics of ***P. liquidambari*** transcriptome**.

Total raw reads	27,401,258
Total clean reads	26,109,074
Total clean nucleotides (nt)	2,349,816,660
Q20 percentage	96.47%
N percentage	0.01%
GC percentage	56.45%
Total number of contigs	51,120
Total length of contigs (nt)	24,882,464
Mean length of contigs (nt)	487
N50 of contigs	1240
Total number of unigenes	32,424
Total length of unigenes (nt)	30,646,255
Mean length of unigenes (nt)	945
N50 of unigenes	1574
Distinct clusters	7,946
Distinct singletons	24,478

### Functional annotation of predicted proteins

**Figure 4:**
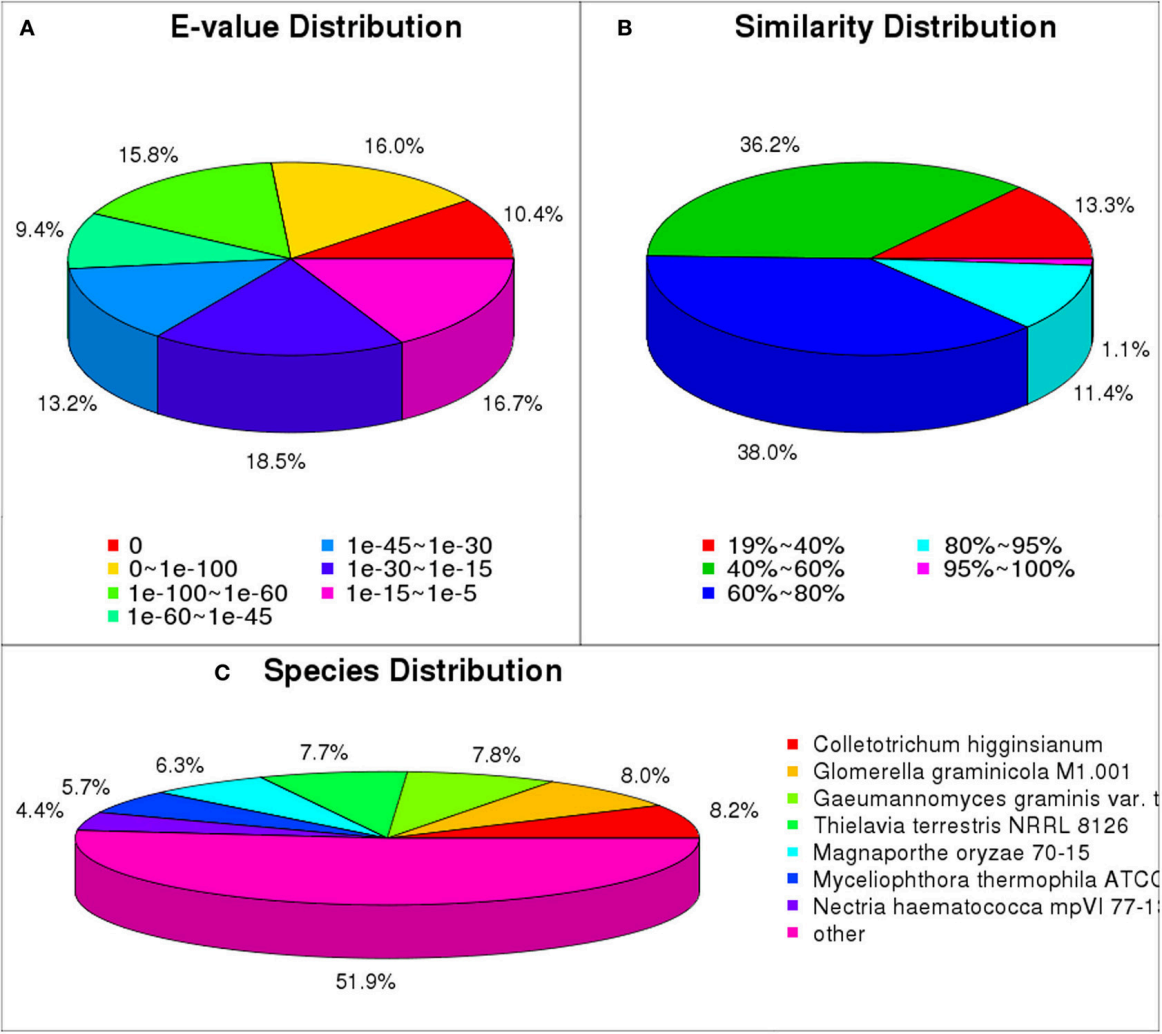
**NR classification of ***P. liquidambari*** unigenes. (A)** *E*-value distribution. **(B)** Identity distribution. **(C)** Species distribution.

We matched unigene sequences against NR, NT, Swiss-Prot, KEGG, GO and COG databases using blastx (*E* < 10^−5^). Of these, 22,700 unigenes (70.0% of total) were annotated, and most could be annotated to protein functional information in the NR database. A total of 22,382 unigenes were annotated to NR database (Table S4). We have analyzed the *E*-value distribution (Figure 4A), similarity distribution (Figure 4B) and species distribution (Figure 4C) of the NR annotation. The *E*-value distribution showed that 64.8% of the mapped sequences displayed a high level of homology (*E* < 10^−30^); 51.6% of the mapped sequences displayed a higher level of homology (*E* < 10^−45^). The similarity distribution has a comparable pattern, with 50.5% of sequences having similarity higher than 60 and 12.5% of the sequences having similarity higher than 80%. For species distribution, 8.2% of the distinct sequences had top matches with sequences from *Colletotrichum higginsianum*, followed by the *Glomerella graminicola* M1.001 (8.0%), *Gaeumannomyces graminis* var. tritici R3-111a-1 (7.8%), *Thielavia terrestris* NRRL 8126 (7.7%), *Magnaporthe oryzae* 70-15 (6.3%), *Myceliophthora thermophila* ATCC 42464 (5.7%), *Nectria haematococca* mpVI 77-13-4 (4.4%) and 48.1% of the unigene sequences matched to the seven species.

### GO and COG classification

**Figure 5:**
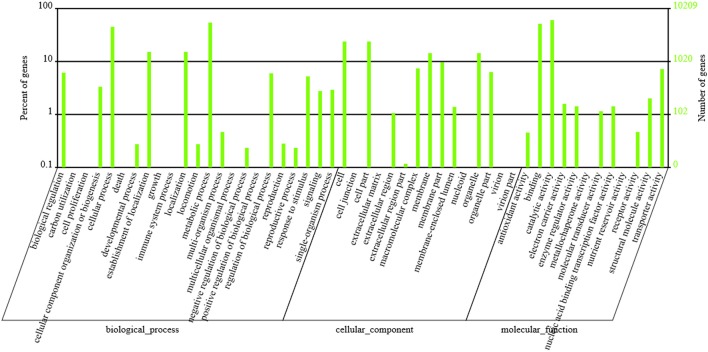
**GO classification of ***P. liquidambari*** unigenes**. Left vertical coordinate indicates the percentage of a specific category of unigenes in the main category, right vertical coordinate indicates the number of unigenes in a category.

GO functional annotation was obtained according to NR annotation information. GO assignments were used to classify the functions of the predicted *P. liquidambari* unigenes. A total of 10,209 unigenes were assigned to 50 functional groups in each of the three main categories according to sequence homology (Figure 5 and Table S5). In the cellular component, the majority was “cell,” “cell part,” “membrane,” “organelle” and “membrane part” unigenes associated with cell membranes and organelles. In the section for molecular function, the dominant functions were “catalytic activity,” “binding,” “transporter activity” and “structural molecule activity.” In the section for biological processes, unigenes were mainly involved in metabolic processes and cellular processes, in agreement with the results of cellular components and molecular function.

**Figure 6:**
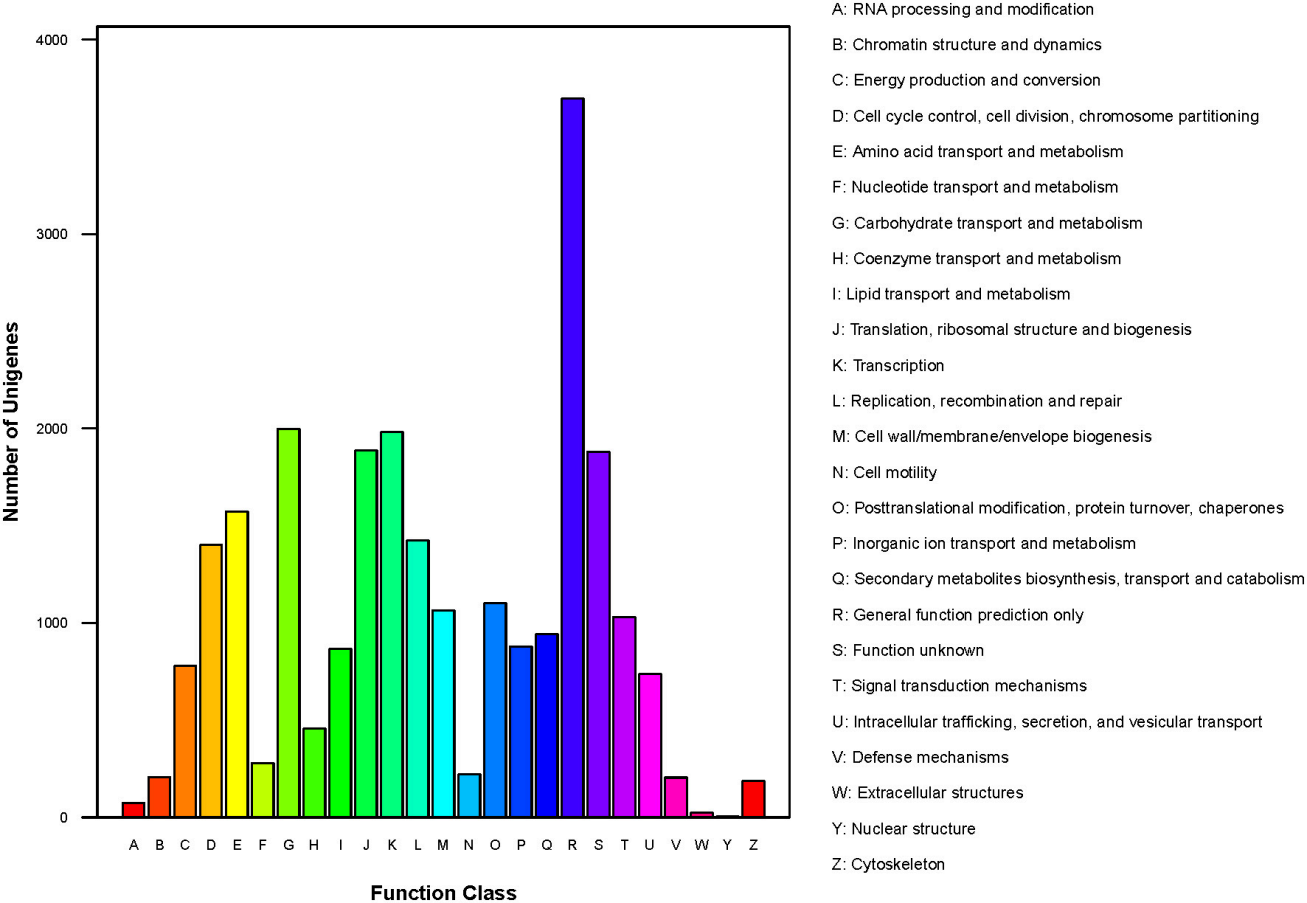
**COG classification of ***P. liquidambari*** unigenes**.

In total, 10,327 unigenes have a COG classification based on sequence homology. Among the 25 COG categories (Figure 6), “general function prediction only” (3698) was the largest group, followed by “carbohydrate transport and metabolism” (1998), “transcription” (1982), and “translation, ribosomal structure and biogenesis” (1887). The groups for “nuclear structure” (5), “extracellular structure” (25) and “RNA processing and modification” (74) were smallest.

### KEGG analysis

A total of 22,700 annotated unigenes of *P. liquidambari* were blasted to the KEGG database and annotated further. In all, 14,791 sequences were found to be involved in 108 signal pathways. The number of sequences ranged from 4 to 4785 (Table S6). The first 20 pathways with the greatest number of sequences are indicated in Table 2, and the pathways that were most represented were metabolic pathways (4785) and biosynthesis of secondary metabolites (2154). These annotations provided important clues for further studying the specific development, function and pathways of *P. liquidambari*. The top 10 metabolic pathways were as follows: starch and sucrose metabolism (1481), amino sugar and nucleotide sugar metabolism (814), purine metabolism (630), pyrimidine metabolism (408), lysine degradation (365), tyrosine metabolism (329), glycolysis/gluconeogenesis (276), fructose and mannose metabolism (249), butanoate metabolism (236) and tryptophan metabolism (226). We believe that genes in carbohydrate metabolism and biosynthesis of secondary metabolites play significant roles in *P. liquidambari* endophytism and saprophytism.

**Table 2:** **List of first 20 pathways with highest sequence numbers**.

**Number**	**Pathway**	**All genes with pathway annotation (14,791)**	**Pathway ID**
1	Metabolic pathways	4785 (32.35%)	ko01100
2	Biosynthesis of secondary metabolites	2154 (14.56%)	ko01110
3	Starch and sucrose metabolism	1481 (10.01%)	ko00500
4	RNA transport	817 (5.52%)	ko03013
5	Amino sugar and nucleotide sugar metabolism	814 (5.5%)	ko00520
6	Purine metabolism	630 (4.26%)	ko00230
7	MAPK signaling pathway - yeast	596 (4.03%)	ko04011
8	Protein processing in endoplasmic reticulum	543 (3.67%)	ko04141
9	Cell cycle - yeast	467 (3.16%)	ko04111
10	mRNA surveillance pathway	445 (3.01%)	ko03015
11	Meiosis - yeast	414 (2.8%)	ko04113
12	Spliceosome	409 (2.77%)	ko03040
13	Pyrimidine metabolism	408 (2.76%)	ko00240
14	RNA degradation	370 (2.5%)	ko03018
15	Lysine degradation	365 (2.47%)	ko00310
16	Tyrosine metabolism	329 (2.22%)	ko00350
17	Endocytosis	281 (1.9%)	ko04144
18	Glycolysis / Gluconeogenesis	276 (1.87%)	ko00010
19	Ribosome biogenesis in eukaryotes	256 (1.73%)	ko03008
20	Fructose and mannose metabolism	249 (1.68%)	ko00051

### Protein coding region (CDS) prediction

In total, 22,300 and 1551 unigenes were predicted by BLASTx and ESTScan, respectively. The histogram seen in Figure S2 shows the length distribution of CDS predicted by BLAST and ESTScan. In general, as sequence length increased, the number of CDS was gradually reduced. This is consistent with unigene assembly results.

### Analysis of differentially expressed genes

**Figure 7:**
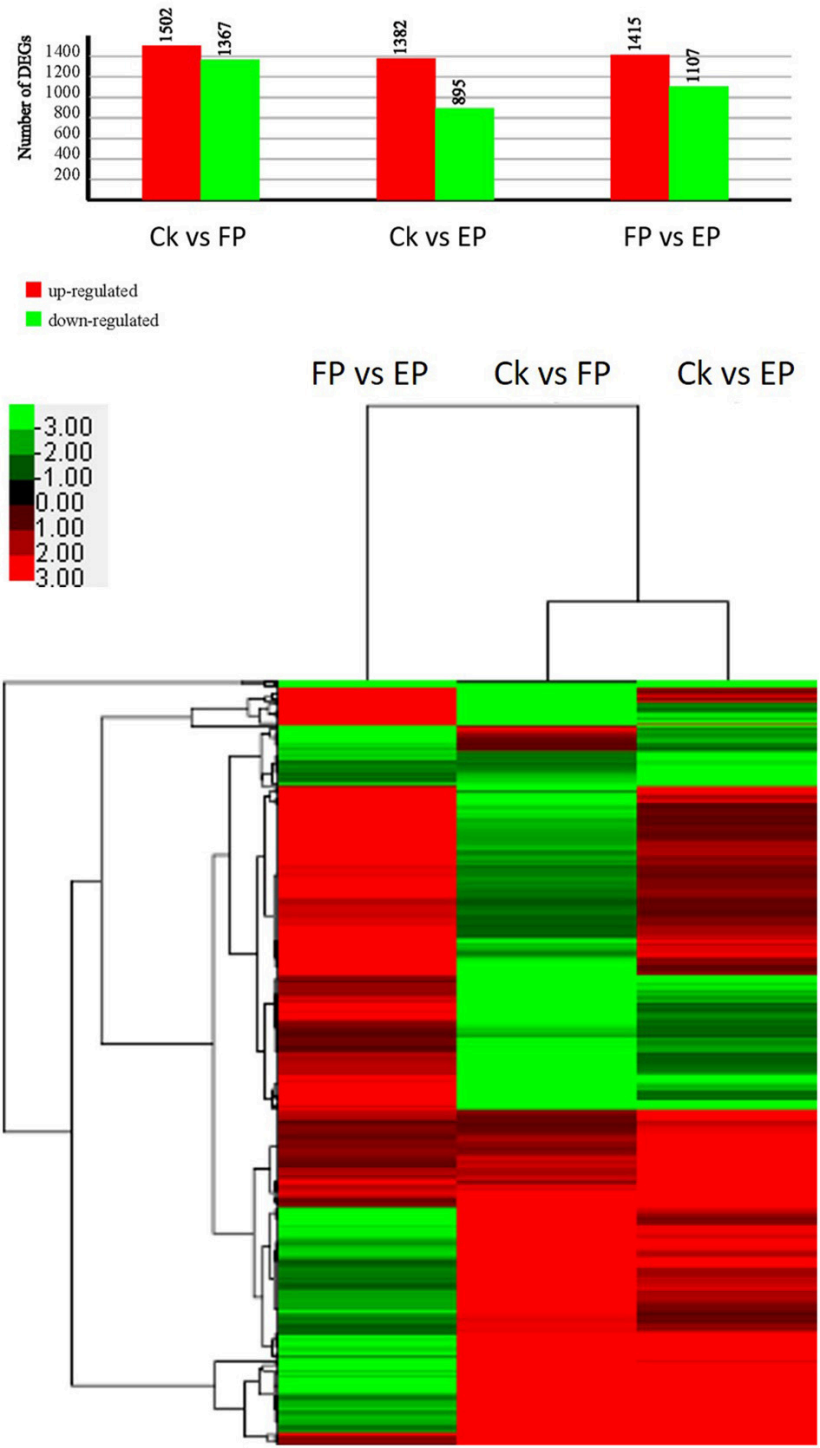
*****P. liquidambari*** DEGs induced under various lifestyles**. Image above, histogram displaying numbers of DEGs for various lifestyles. Image below, clustering of co-expressed genes among three expression patterns; green to red represent gene expression levels.

To detect the DEGs between EP and FP, we screened differentially expressed tags between samples according to the method described by Audic and Claverie (1997). As shown in Figure 7, there were 2869 genes that were differentially expressed between Ck and FP. Among these genes, 1502 were up-regulated and 1367 were down-regulated in response to the FP switch. There were 2277 genes differentially expressed between Ck and EP. Among these genes, 1382 genes were up-regulated and 895 were down-regulated in response to the EP switch. There were 2522 genes differentially expressed between FP and EP. Among these genes, 1415 genes were up-regulated and 1107 were down-regulated in response to the switch between EP and FP. There were 491 genes co-expressed among the three expression patterns (Figure 7). DEGs were further categorized into different functional groups by GO and KEGG pathway enrichment analysis. Compared with Ck, “starch and sucrose metabolism” and “amino sugar and nucleotide sugar metabolism” were the most enriched pathways in FP, and “butanoate metabolism” was the most enriched pathway in EP. Compared with FP, the “ribosome” group was the most enriched pathway in EP (*P* < 0.05) (Table S7).

### Validation of RNA-seq data by qPCR

**Figure 8:**
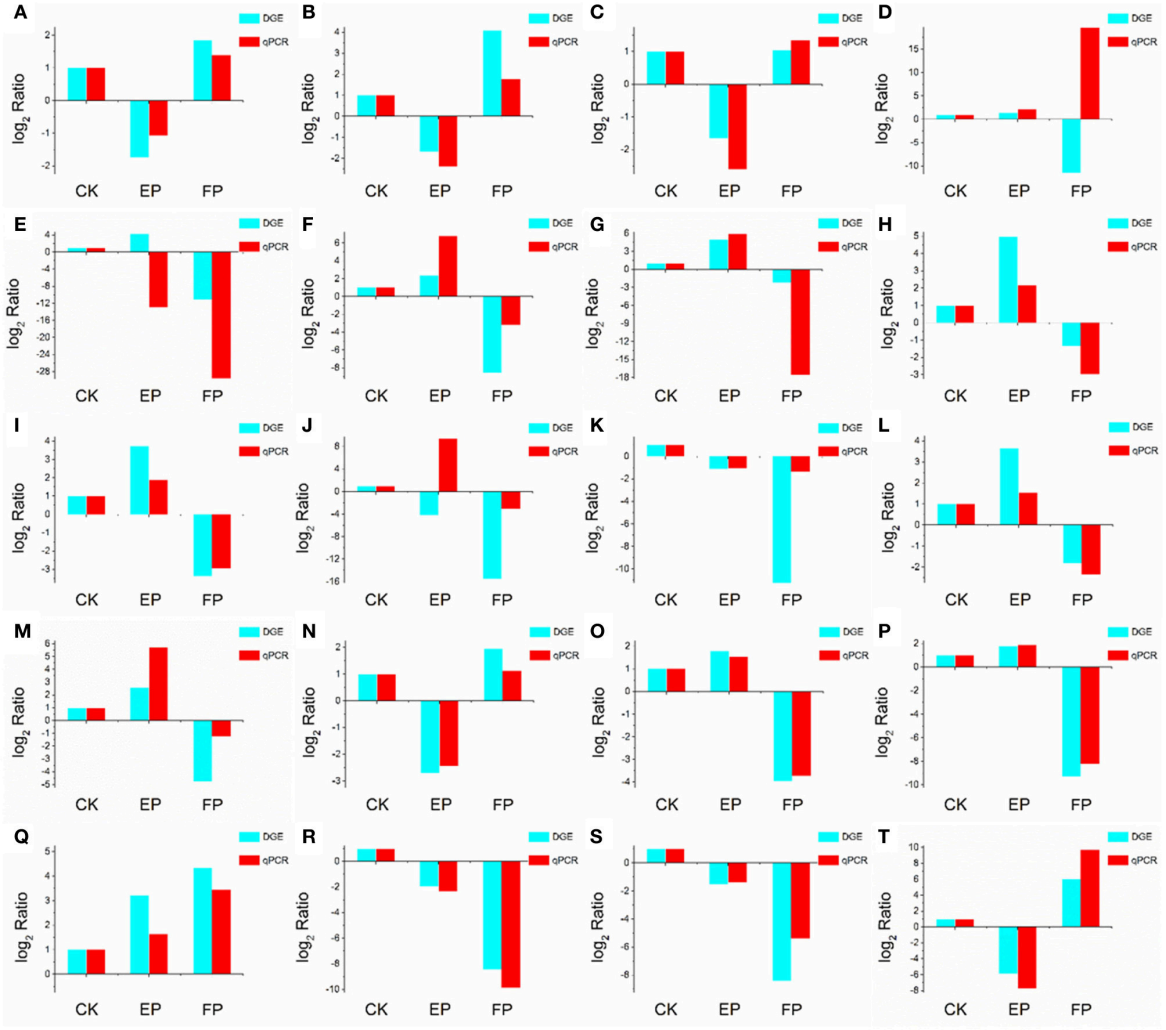
**Confirmation of DEGs by qRT-PCR**. The x-axis indicates treatment method. The y-axis indicates relative expression level. The following genes were tested (with description). **(A)** glutaminase a (Unigene11948_A), **(B)** probable lysosomal cobalamin transporter (Unigene16976_A), **(C)** NAD(P)-binding protein (Unigene17203_A), **(D)** putative alcohol dehydrogenase (Unigene6694_A), **(E)** cytochrome P450 monooxygenase (Unigene14342_A), **(F)** beta-glucosidase (Unigene19402_A), **(G)** pyruvate decarboxylase (Unigene13421_A), **(H)** cyanide hydratase (Unigene13743_A), **(I)** major facilitator superfamily transporter (CL403.Contig1_A), **(J)** glycoside hydrolase family 72 protein (Unigene7006_A), **(K)** transmembrane amino acid transporter (Unigene19132_A), **(L)** epl1 protein (Unigene2645_A), **(M)** heavy metal translocating *P*-type ATPase (Unigene13596_A), **(N)** glutathione S-transferase Gst3 (Unigene2773_A), **(O)** phosphoadenosine phosphosulfate reductase (Unigene7219_A), **(P)** nucleolar protein nop-58 (CL1651.Contig1_A), **(Q)** laccase-like protein (Unigene11698_A), **(R)** high affinity copper transporter (Unigene9769_A), **(S)** 3-ketoacyl-CoA reductase (Unigene3887_A), **(T)** glucan 1,4-alpha-maltohexaosidase (Unigene17276_A).

To validate the DEGs obtained by Solexa RNA-seq, we further performed quantitative real-time PCR analysis on 20 representative genes involved in the three lifestyles (Figure 8). We found that fold-change values of most DEGs using real-time qRT-PCR exhibited trends similar to RNA-Seq samples. Differential expression was observed for all candidate genes, suggesting that they are involved in regulatory networks that are active during the three environmental conditions. Only three genes (putative alcohol dehydrogenase, cytochrome P450 monooxygenase and glycoside hydrolase family 72 protein) did not show consistent expression between qRT-PCR and RNA-seq data sets. Comparison of data from Solexa sequencing analysis methods with data obtained from qRT-PCR indicates high credibility for these sequencing methods.

## Discussion

### Colonization of *P. liquidambari* in rice

To investigate the fate and behavior of *P. liquidambari* in rice *in situ*, B3 was tagged with the *gfp* gene. Colonization patterns of *P. liquidambari* were roughly divided into three successive time-space stages. First, extracellular colonization of runner hyphae outside the root, mainly concentrated in the base of the root hair, gradually formed a hyphal network on the root surface (<3 dai) (Figures 1A–F and Video S1). Next, entering the biotrophic phase, intracellular and extracellular hyphae underwent branching growth along the root axis, and hyphae were extruded in a deformed fashion (4–10 dai) (Figures 1H–M), meaning that fungal infection began to be restricted. Finally, in the stage of colonization associated with programmed cell death, the vast majority of epidermal cells and part of the outer cortex cells were crowded with a large number of mycelium and sclerotium; the fungal structure indicated that fungal infection was further blocked (>15 dai) (Figures 1S,T). Interestingly, *P. liquidambari* still colonized when the host aged or died (Figures 1U–W). It is possible that *P. liquidambari* activated saprophytic programs to adapt to this variation. Colonization patterns of *P. liquidambari* in rice were different from *Harpophora oryzae* (Su et al., 2013), basidiomycete endophyte *Piriformospora indica* (Zuccaro et al., 2011; Lahrmann et al., 2013), soil invaders *Fusarium equiseti*, and *Pochonia chlamydosporia* (Maciá-Vicente et al., 2009), which belong to strict root endophytes. It was similar to *Colletotrichum tofieldiae*, in that a fraction of strong hypha penetrated and entered the root axis center, through the vascular bundle into the acrial part (Hiruma et al., 2016). During root infection, *P. liquidambari* generated a fungal structure similar to phytopathogen, with neck-like constriction (Figure 1N), showing that any phytopathogen or endophyte can form similar infection structures during root infection. This phenomenon is related to infected tissue and thus belongs to tissue-specific infection. The most obvious similarity between *P. liquidambari* and phytopathogens is that they can infect vascular tissues and systematically spread to acrial parts through vascular tissue (Figures 1O–R).

In the colonization process of *P. liquidambari*, a large number of hyphae were limited to the epidermal layer and rhizosphere, and only a fraction of hyphae penetrated to the cortex. This fully demonstrates that *P. liquidambari* colonization is restricted by space and quantity. In late infection, *P. liquidambari* biomass remained at a steady state after hyphae entered the cortex cells, which can be explained by a reproduction rate of endophyte that was controlled within a certain range (Figure 2). In contrast, the reproduction of pathogen in the root was unrestricted and spread from roots to acrial parts, increasing biomass in an unrestricted fashion and inducing plant disease (Marcel et al., 2010; Su et al., 2013). In contrast, with this exploding pathogenic infection, the biomass of *P. liquidambari* in the host was maintained in an appropriate range without excessive reproduction. This is reminiscent of endophyte *H. oryzae* symbiosis with rice, initially showed moderate proliferation, subsequently colonization increased rapidly, finally reaching a steady-state level in rice roots (Su et al., 2013). Endophyte *C. tofieldiae* biomass was significantly increased in Trp-derived metabolites mutant, resulted in a severe negative effect on the growth of this mutant and eventually killed the plants (Hiruma et al., 2016). Likewise, the increased colonization of indolic glucosinolates mutant by root-associated fungi *P. indica* and *Sebacina vermifera* led in turn to plant death, suggesting compromised mutualism (Lahrmann et al., 2015). Therefore, another important difference between hostile interactions and mutualistic interactions is quantity rather than quality.

### Endophytic and saprophytic systems and transcriptome sequencing of *P. liquidambari*

The transcriptome is the subset of genes active in tissues and species. To understand the dynamic of the transcriptome it is key to explain the phenotypic changes caused by combinations of genotype and environmental factors (Rockman and Kruglyak, 2006). Recently, Illumina RNA-seq has been used to identify genes of microbes related to plant interactions (Kawahara et al., 2012; O'connell et al., 2012; Alkan et al., 2015). RNA-seq can be used not only to detect organismic transcripts in existing genomic sequences but also to sequence non-model organisms lacking genomic information. To our knowledge, this is the first study of *Phomopsis* sp. using RNA-seq technology to obtain whole transcriptome information. Our experimental results provide more resources and sequences for studying filamentous endophyte *P. liquidambari*.

In the past, researchers have investigated the molecular genome of endophytes *in vivo*, but are challenged in retrieving endophyte gene information from high genetic background from the host plant. Compared to unrestrained pathogen reproduction after infection, endophytes steadily undergo symbiosis with the host and establish a subtle counterbalancing relationship that largely limits endophyte growth in the host. When host plants are used as vectors for transcriptome level research, a small number of expressed endophyte genes are typically disregarded due to the significant background of plant genes (Porras-Alfaro and Bayman, 2011). The callus is an undifferentiated living cell structure of plants that contains a set of defense systems similar to host plants (Nawrot-Chorabik et al., 2016). In this study, we used dual cultures of rice callus and *P. liquidambari* to simulate an endophytic environment in which organisms can release signals to recognize each other and form a relationship of simulated antagonism balance. Fungi growth on the callus surface can provide fungal hyphae directly, avoiding the interference from host cell. In litter culture, the humic acid substances of litter will significantly affect RNA quality. Thus, we adopted a litter liquid culture to suspend fungi in a liquid, collected the hyphae by flushing with sterile water and extracted RNA. The quality was improved using this method. All transcriptome sequencing items were fit with the measurable indicators; Q20 percentage >80%, N percentage <0.5% and GC percentage was 35–65%, showing that the sequencing output and sequence quality were of good quality and could be further analyzed (Table 1).

### Divergent expression patterns from amino acid metabolism to fatty acid biosynthesis

**Figure 9:**
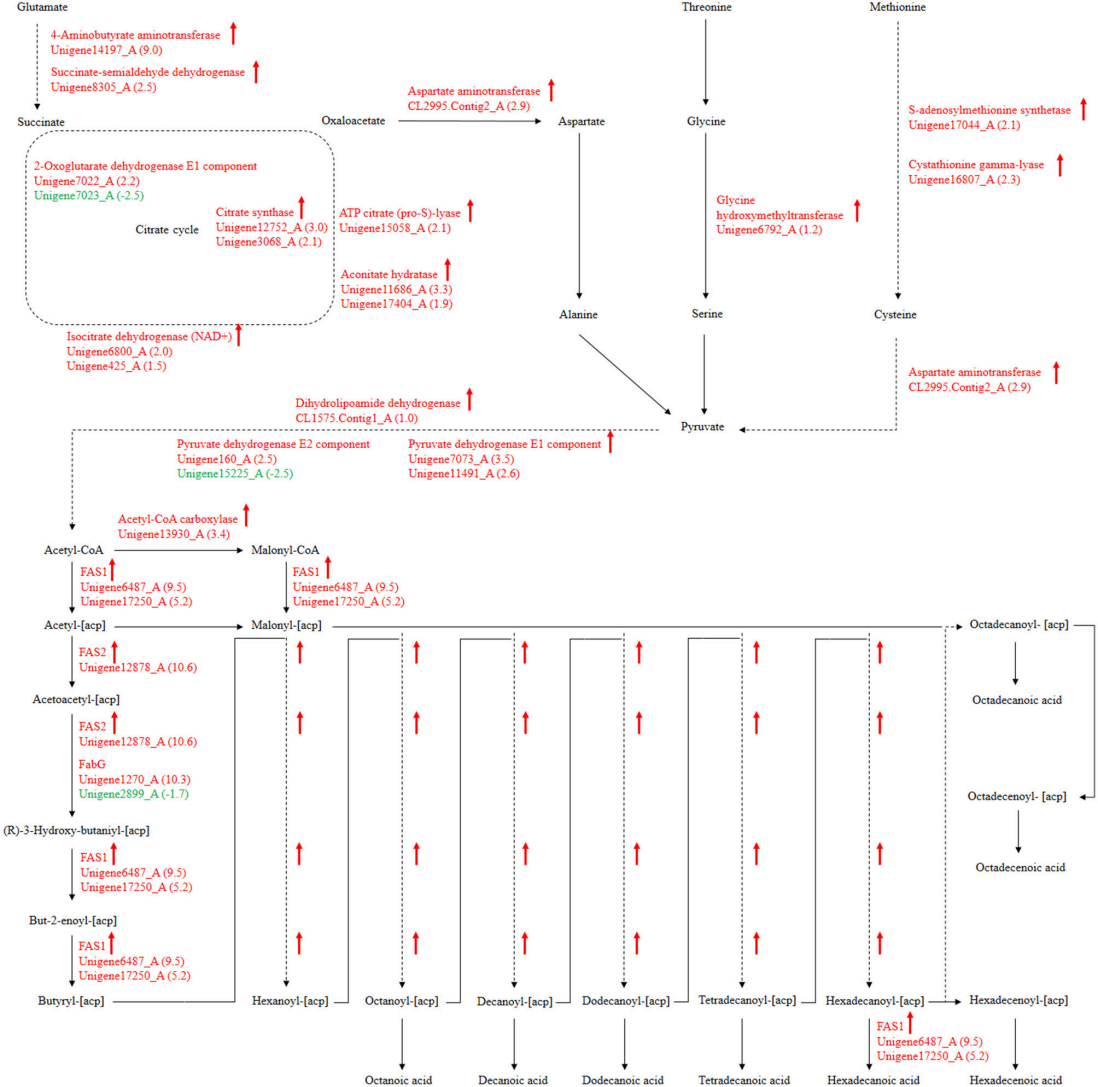
*****P. liquidambari*** significantly up-regulated pathways leading to fatty acid biosynthesis from amino acids metabolism and carbohydrate metabolism in FP vs. EP**. Red represents up-regulated transcripts and green represents down-regulated transcripts.

For our research, 108 biological pathways including the starch and sucrose metabolism pathway, amino sugar and nucleotide sugar metabolic pathways, the fatty acid biosynthesis pathway, and many others were identified by KEGG pathway analysis of unigenes. A total of 1638, 1330, and 1503 DEGs with pathway annotations were identified in the three respective contrast groups. From those pathways, we selected the fatty acid biosynthesis pathway, which is connected to amino acid metabolism and involved in carbohydrate metabolism, for deep analysis. The citrate cycle is the key pathway to energy metabolism. For *P. liquidambari* in the EP compared with FP, genes of the citrate cycle were differentially expressed, significantly enriched (*P* < 0.05), and nearly up-regulated (Figure 9 and Table S7). Most genes for oxidative phosphorylation related to the citrate cycle were also up-regulated. Carbon flux in the fatty acid biosynthesis pathway not only determines the component but also the content of fatty acids in fungi (Hao et al., 2014). Because alanine, aspartate and glutamate metabolism are closely connected to the citrate cycle, and thus genes for these pathways are also enriched and mostly up-regulated. The genes of glycine, serine and threonine metabolism and cysteine and methionine metabolism were up-regulated. The common product of these amino acids metabolisms is pyruvate, and a supply of acetyl-CoA plays a more important role in fungal fatty acid biosynthesis. In *Mortierella alpina*, tyrosine and phenylalanine were considered to contribute NADPH and acetyl-CoA to lipid metabolism through a phenylalanine-hydroxylating system (Wang et al., 2013).

Fatty acid synthesis and transformation plays an important role in fungal growth and development. During intraradical growth, much fatty acid synthesis is required for lipid storage and membrane proliferation of fungi. Adaptation of lipid metabolism may be the prerequisite for symbiosis to achieve function compatibility between fungi and periarbuscular membrane. Fungi were forced to change their membrane lipid composition to allow nutrient exchange between fungal arbuscular and plant periarbuscular membranes (Wewer et al., 2014). In the EP, compared to FP, nine DEGs associated with fatty acid biosynthesis were co-expressed. Interestingly, two identified unigenes were identical to those identified from the GO analysis. Among those nine DEGs, only one unigene (Unigene2899_A) was down-regulated; this unigene encodes 3-oxoacyl-[acyl-carrier protein] reductases (FabG). The other eight unigenes were up-regulated and may be positively regulated genes in the fatty acid biosynthesis pathway. Figure 9 shows the locations of DEGs in the fatty acid biosynthesis pathway. The many up-regulated genes indicate that more positively controlled genes than negatively regulated genes function in fatty acid biosynthesis.

Polyunsaturated fatty acids or oxylipins can trigger extensive cellular responses, such as pathogenicity arsenals, defense and stress response, secondary metabolism, oxylipin synthesis and cell wall formation. This indicates that generation and recognition are important for coordinating these responses, which can guide pathogen adaptation to host response (Tsitsigiannis and Keller, 2007). Fungal oxylipin repertoire may participate in the competition between pathogen and host and is also involved in reproduction and development (Oliw et al., 2016). Recent evidence has showed that fatty acids also appear and play a role in beneficial plant-fungi interactions. The endophyte *Fusarium incarnatum* in the embryo of *Aegiceras corniculatum* can produce archetypal plant defense oxylipins that can protect the embryo and are derived from linoleic acid (Pohl and Kock, 2014). Esterified fatty acids of *Lasiodiplodia theobromae* can be used as plant growth regulators in tobacco and have similar activity to gibberellic acid (Uranga et al., 2016). In addition, commensal *Candida albicans* produce a low-level of resolvin E1, an eicosanoids that works as an effective anti-inflammatory lipid and can inhibit adaptive immune responses and protect commensal yeast from host immune attacks (Pohl and Kock, 2014). DGE data show that genes encoding the FAS1, FAS2, and Acetyl-CoA carboxylase of fatty acid synthesis were up-regulated in EP. FabG genes were both up- and down-regulated (Figure 9). There are considerable differences in fatty acid synthesis between the symbiotic and asymbiotic states. Lipid compounds play a key role in symbiotic signals and are likely involved in signaling communication between plants and endophytes. Oxidized fatty acids as signaling molecules have an ancient evolutionary origin and are ideal candidates for inter-kingdom signaling communication (Pohl and Kock, 2014). Oxylipins as intracellular and intercellular communication signals showed vital bioactivities in fungi, plants and animals. The oxylipin signature profile of fungi serves an adjusting function as a “master switch” under different environmental conditions and provides the appropriate mechanisms to microbes by balancing meiospore and mitospore development temporarily. On the basis of *Aspergillus*–seed pathosystems, as supported by data, oxylipin cross-talk is reciprocal. The structural similarity of plant and fungal oxylipins has given rise to a hypothesis that they are important molecules in cross-kingdom communication (Tsitsigiannis and Keller, 2007).

### Differences in secondary metabolism from terpenoid to steroid biosynthesis

**Figure 10:**
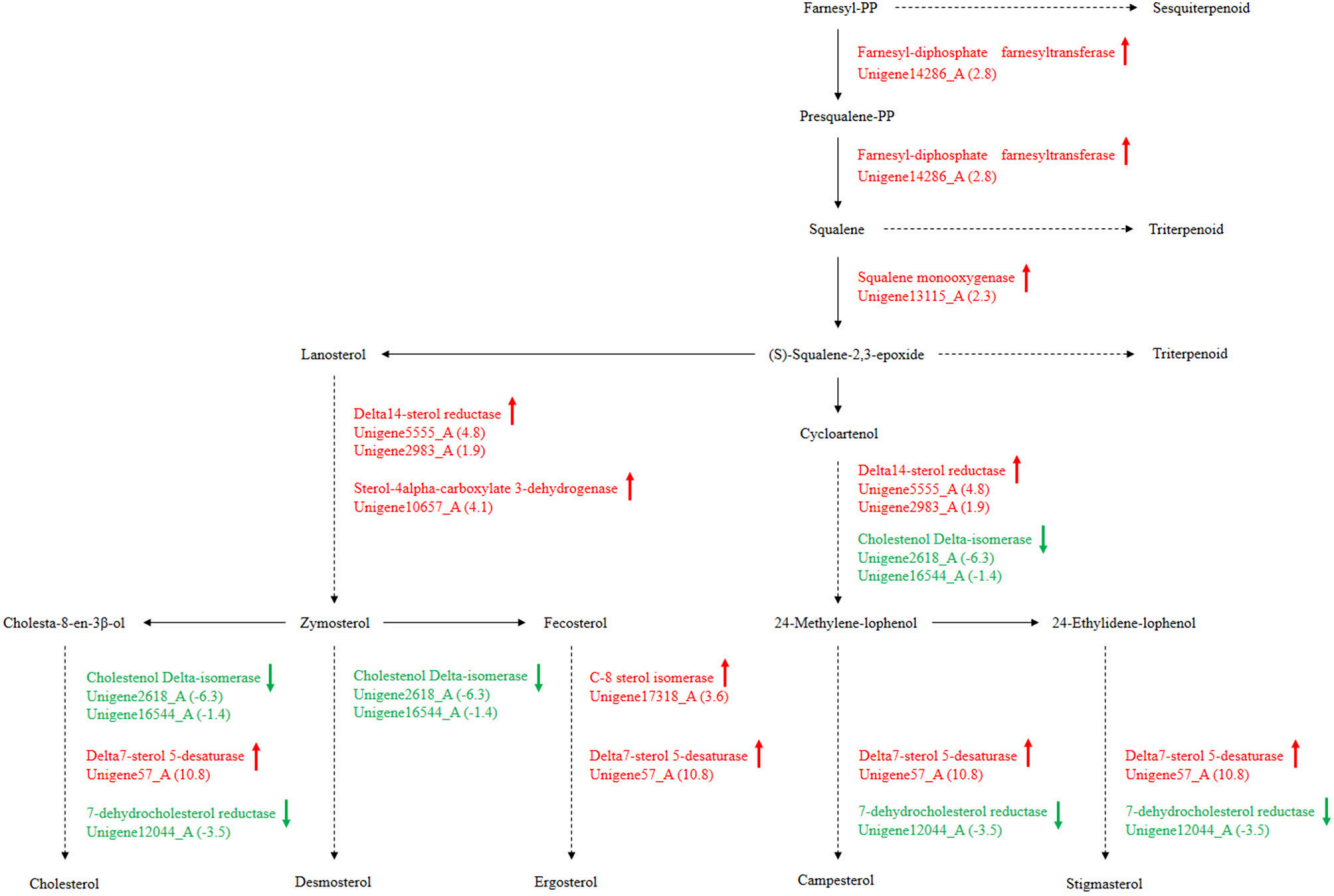
*****P. liquidambari*** secondary metabolism from terpenoid to steroid biosynthesis in FP vs. EP**. Red represents up-regulated transcripts, green represents down-regulated transcripts and black represents unchangeable transcripts.

Secondary metabolism is strictly regulated by fungi and is often closely related to asexual reproduction. Secondary metabolites produced from *Trichoderma* include pyrone, antimicrobial peptides and terpenoids that can inhibit the growth of phytopathogens. Several studies have reported that endophytes are involved in the synthesis of plant secondary metabolites during symbiosis with plants. For example, as *Phoma Medicaginis* switches from the endophytic stage to the saprophytic stage, a large increase in the production of brefeldin A contributes to host defense competitive saprophytes. Low levels of these compounds will inhibit the defense system to maintain the endophytic state of *P. Medicaginis* (Weber et al., 2004). Endophytes can affect growth processes by influencing secondary metabolism under different habitats. Genes encoding secondary metabolism in *Epichloe* spp., *C. tofieldiae* and *H. oryzae* were significantly expressed, but strongly reduced in sebacinales, indicating convergent adaptation to a life inside living host cells (Zuccaro et al., 2011; Fesel and Zuccaro, 2016). Cytochrome P450 monooxygenases play diverse and vital roles in various metabolisms and when fungi adapt to specific ecological niches (Chen et al., 2014). As shown in Table 3, compared with FP, the corresponding genes of secondary metabolites synthesis pathways (e.g., stilbenoid, diarylheptanoid and gingerol biosynthesis, phenylpropanoid biosynthesis, terpenoid backbone biosynthesis, ubiquinone and other terpenoid-quinone biosynthesis), were up-regulated. Most of these genes coded for the cytochrome P450 enzyme family and revealed that various cytochrome P450s are involved when filamentous fungi generate a large number of secondary metabolites. Similarly, a phylogenetic analysis revealed the specific expansion of secondary metabolite synthesis genes in *H. oryzae*, as well as cytochrome P450 monooxygenases (Xu et al., 2014). Cytochrome P450 catalyzes biosynthetic metabolism of endogenous substances with important physiological functions such as fatty acids, terpenoids and hormones, and thus P450 plays an important role in the modification of secondary metabolites. In tryptophan metabolism, tryptophan is converted into indole derivatives through these cytochrome P450 enzymes and further forms various secondary metabolites. Several secondary metabolisms originating from tryptophan were essential for beneficial symbiosis with *C. tofieldiae*. Mutation of the organism not only ended this beneficial symbiotic relationship, but increased colonization of *C. tofieldiae* such that it eventually killed the host plant (Hiruma et al., 2016). DGE showed that the corresponding genes in the pathway of sesquiterpenoid and triterpenoid biosynthesis were significantly up-regulated (*P* < 0.05) (Figure 10 and Table S7). This is consistent with a previous study that shoed that endophyte *Gilmaniella* sp. AL12 can establish symbiosis with *Atractylodes lancea* and greatly promotes terpenoids accumulation in the herb (Yuan et al., 2016). We found two up-regulated genes in EP: farnesyl-diphosphate farnesyl transferase (Unigene14286_A), involved in isoprenoid biosynthesis, and squalene monooxygenase (Unigene13115_A). Both enzymes have oxidoreductase activity and effect secondary metabolites synthesis and plant-endophyte symbiosis. They also participated in steroid biosynthesis in lipid metabolism. Cytochrome P450 also plays housekeeping functions in fungi. For instance, CYP51 takes part in sterol biosynthesis and is a popular antifungal target to control fungal disease in humans and crops (Becher and Wirsel, 2012). Previous studies have shown that CYP51 and CYP61 play housekeeping functions in the sterol biosynthesis of filamentous fungi (Kelly et al., 2009). The personalized cytochrome P450 components of fungi indicate that highly specialized functions enable evolutionary adaptation to ecological niches.

**Table 3:** **Upregulation of genes involved in biosynthesis of secondary metabolites in FP vs. EP**.

**Category or unigene ID**	**Gene ID**	**Annotation**	**Accession no.**	**Log_2_ratio**
Unigene14342_A	145246720	cytochrome P450 monooxygenase	XP_001395609.1	15.43
Unigene113_A	310795890	cytochrome P450	EFQ31351.1	14.26
Unigene13908_A	302415555	cytochrome P450 monooxygenase	XP_003005609.1	14.20
Unigene6720_A	310789565	oxidoreductase NAD-binding domain-containing protein	EFQ25098.1	12.81
Unigene10987_A	322705062	branched-chain-amino-acid aminotransferase	EFY96651.1	12.29
Unigene3485_A	16416002	probable cytochrome P450 monooxygenase (lovA)	CAB91316.2	12.04
Unigene19402_A	302423734	beta-glucosidase	XP_003009697.1	10.93
Unigene2980_A	347839081	similar to cytochrome P450 monooxygenase	CCD53653.1	10.88
Unigene57_A	52548220	C-5 sterol desaturase C-like	AAU82099.1	10.77
Unigene14360_A	400600304	Cytochrome P450 CYP505D4	EJP67978.1	10.50
Unigene10045_A	380493536	trichothecene C-8 hydroxylase	CCF33809.1	10.47
Unigene1270_A	358369448	3-ketoacyl-acyl carrier protein reductase	GAA86062.1	10.34
Unigene19132_A	380492609	transmembrane amino acid transporter	CCF34481.1	10.13
Unigene7296_A	240282093	cytochrome P450	EER45596.1	9.89
Unigene17476_A	380488167	CTP synthase	CCF37564.1	9.41
Unigene19143_A	380479107	benzoate 4-monooxygenase cytochrome P450, partial	CCF43218.1	8.47

### Distinct xenobiotic biodegradation and metabolism

Our previous studies have reported the capacity of *P. liquidambari* to decompose phenolic acids, cellulose, N-heterocyclic indole and the polycyclic aromatic hydrocarbon phenanthrene (Dai et al., 2010a,b; Chen et al., 2011, 2013b,c). The cytochrome P450 family also contributes to ecological functions as a decomposer or saprophyte. For example, the cytochrome P450s of white-rot fungi *Phanerochaete chrysosporium* is involved in vast xenobiotic biodegradation of extensive environmental toxic chemicals and the natural aromatic polymer lignin (Syed and Yadav, 2012). The diversity of cytochrome P450s may be closely related to fungal survival environment. For example, the cytochrome P450s of white-rot and brown-rot fungi break down plant materials in the environment (Eastwood and Watkinson, 2011; Chen et al., 2014). As DGE showed that cytochrome P450s are involved in xenobiotic biodegradation, we speculated that endophyte *P. liquidambari* appropriately biodegraded harmful xenobiotics or used them as carbon sources to adapted to the host after entering. In addition, endophytes promote production of secondary metabolites that are beneficial to the host and enable both the endophytes and host plants both to grow. However, endophytes affected by defensive responses and host plant metabolites cannot overgrow at large scales, resulting in a subtle symbiotic relationship.

It has been increasingly demonstrated that endophytes that quickly decompose plant litter *in vitro* can initiate saprophytic effects when the endophytic survival environment is destroyed, for example through plant senescence or falling. This saprophytic effect is similar to a saprophytic lifestyle that maintains survival and growth by metabolizing compounds in litter that are usually difficult to decompose. In this study, we found that the expression of partial genes involved in xenobiotic biodegradation and metabolism of *P. liquidambari* in FP was up-regulated by DGE. As shown in Table 4, in EP compared with FP, genes of pathways concerning bisphenol degradation, chloroalkane and chloroalkene degradation, caprolactam degradation, polycyclic aromatic hydrocarbon degradation, naphthalene degradation, chlorocyclohexane and chlorobenzene degradation, aminobenzoate degradation, styrene degradation, fluorobenzoate degradation, atrazine degradation, dioxin degradation, toluene degradation, benzoate degradation, ethylbenzene degradation, metabolism of xenobiotics by cytochrome P450 and drug metabolism by cytochrome P450 were both up- and down-regulated. This indicates that *P. liquidambari* can decompose heterocyclic compounds in both endophytic and saprophytic lifestyles. However, most genes are down-regulated in FP vs. EP, indicating that the ability of *P. liquidambari* to degrade aromatic or phenolic compounds was enhanced in a saprophytic lifestyle. Endophyte *P. liquidambari* in a simulated saprophytic environment that lacked nutrition that can be utilized directly forced the fungus to decompose residual organic matter in the litter. Lignin, a main component of litter, was also utilized by the fungus because the corresponding genes for biodegradation of xenobiotics such as bisphenol via lignin degradation were up-regulated. This further verified previous research by Dai et al. (2010b), who found that endophytes can decompose polycyclic aromatic hydrocarbons *in vitro*. Chen et al. (2013a) reported that application of endophyte *P. liquidambari* to soil observably promoted the release of inorganic nitrogen through organic matter degradation. Co-culture of *P. liquidambari* with indole and litter increased indole degradation significantly: 99.1% of indole was removed after 60 h of cultivation, and residual indole levels were below the detection threshold at the 84 h time point (Chen et al., 2013c). Zhou et al. (2014a,b) utilized food waste and wheat straw as nutrient sources in a simulated saprophytic system of cultured *P. liquidambari*. The fermentation product was applied to continuously cropped peanut soil, and the concentrations of vanillic acid, coumaric acid, and 4-hydroxybenzoic acid in soil had decreased by 52.5, 49.4, and 57.4%, respectively, after 28 days. The bacterial and fungal community structures in the rhizosphere soil were affected by changes in phenolic acid concentration and promoted peanut seedling growth and nodulation. These changes demonstrate the application prospects for *P. liquidambari* in the decomposition of difficult-to-decompose organic compounds and environmental remediation. In addition, the results indicate the advantages of nutrient restoration to successive cropping of farmland, when plant residue exempt from plowing can thoroughly decompose.

**Table 4:** **Expression profiles of pathways involved in xenobiotics biodegradation in FP vs. EP**.

**Enzyme name**	**Orthology**	**Numbers of unigene**	**Numbers of up-regulated gene**	**Numbers of down-regulated gene**	**Expression**
**BISPHENOL DEGRADATION**					
1.14.-.-	K00517	23	14	9	Up
4.2.1.-	K01726	1	0	1	Down
1.14.13.-	K00492	4	2	2	
1.13.-.-	K05915	1	0	1	Down
1.1.1.-	K00100	9	1	8	Down
3.1.1.-	K01066	2	1	1	
**CHLOROALKANE AND CHLOROALKENE DEGRADATION**					
1.1.1.1	K13953; K00121	6	1	5	Down
1.2.1.3	K00128	1	0	1	Down
3.8.1.-	K01564	2	1	1	
1.1.1.-	K00100	9	1	8	Down
3.8.1.2	K01560	1	1	0	Up
**CAPROLACTAM DEGRADATION**					
1.14.13.22	K03379	1	0	1	Down
3.1.1.17	K01053	1	0	1	Down
1.1.1.2	K00002	2	0	2	Down
**POLYCYCLIC AROMATIC HYDROCARBON DEGRADATION**					
1.3.1.-	K00224	3	0	3	Down
1.14.-.-	K00517	23	14	9	Up
1.14.13.1	K00480	4	1	3	Down
1.14.13.-	K00492	4	2	2	
2.1.1.-	K00599	2	1	1	
**NAPHTHALENE DEGRADATION**					
1.14.13.-	K00492	4	2	2	
1.14.13.1	K00480	4	1	3	Down
1.13.-.-	K05915	1	0	1	Down
4.2.1.-	K01726	1	0	1	Down
1.2.1.-	K00155	1	0	1	Down
1.1.1.1	K13953; K00121	6	1	5	Down
**CHLOROCYCLOHEXANE AND CHLOROBENZENE DEGRADATION**					
3.1.1.45	K01061	3	1	2	
1.14.13.-	K00492	4	2	2	
3.8.1.2	K01560	1	1	0	Up
1.14.13.7	K03380	1	0	1	Down
1.3.1.-	K00224	3	0	3	Down
**AMINOBENZOATE DEGRADATION**					
3.5.1.4	K01426	1	0	1	Down
1.14.13.12	K07824	3	3	0	Up
1.14.-.-	K00517	23	14	9	Up
1.14.13.7	K03380	1	0	1	Down
3.1.1.-	K01066	2	1	1	
3.1.3.1	K01113; K01077	3	0	3	Down
3.1.3.2	K01078	1	0	1	Down
1.14.13.-	K00492	4	2	2	
2.3.1.-	K00680	5	4	1	Up
1.14.14.1	K14338; K00493	2	1	1	
**STYRENE DEGRADATION**					
3.5.1.4	K01426	1	0	1	Down
1.13.11.5	K00451	1	0	1	Down
3.7.1.2	K01555	1	0	1	Down
**FLUOROBENZOATE DEGRADATION**					
3.1.1.45	K01061	3	1	2	
**ATRAZINE DEGRADATION**					
3.8.1.-	K01564	2	1	1	
**DIOXIN DEGRADATION**					
1.14.13.1	K00480	4	1	3	Down
**TOLUENE DEGRADATION**					
1.14.13.7	K03380	1	0	1	Down
1.14.13.-	K00492	4	2	2	
3.1.1.45	K01061	3	1	2	
**BENZOATE DEGRADATION**					
1.14.13.12	K07824	3	3	0	Up
4.2.1.-	K01726	1	0	1	Down
2.3.1.16	K00632	1	1	0	Up
2.3.1.-	K00680	5	4	1	Up
2.3.1.9	K00626	1	0	1	Down
**ETHYLBENZENE DEGRADATION**					
2.3.1.16	K00632	1	1	0	Up
2.3.1.-	K00680	5	4	1	Up
**METABOLISM OF XENOBIOTICS BY CYTOCHROME P450**					
3.3.2.9	K01253	1	0	1	Down
2.5.1.18	K00799	1	1	0	Up
1.1.1.1	K13953; K00121	6	1	5	Down
1.2.1.5	K00129	1	1	0	Up
**DRUG METABOLISM - CYTOCHROME P450**					
1.14.13.8	K00485	5	1	4	Down
2.5.1.18	K00799	1	1	0	Up
1.1.1.1	K13953; K00121	6	1	5	Down
1.2.1.5	K00129	1	1	0	Up

## Author contributions

JZ performed most of the work, including experimental design and operation, data analysis and manuscript writing. XL and YC prepared samples and extracted fungal RNA. CD supervised all work.

### Conflict of interest statement

The authors declare that the research was conducted in the absence of any commercial or financial relationships that could be construed as a potential conflict of interest.

## Supplementary material

Table S1 **qPCR primer sequences**.

Table S2 **Length distribution of contigs**.

Table S3 **Length distribution of unigenes**.

Table S4 **Functional annotation of unigenes**.

Table S5 **GO classification of P. liquidambari unigenes**.

Table S6 **KEGG annotation of ***P. liquidambari*** transcriptome**.

Table S7 **Significantly enriched pathway in Ck vs. EP, Ck vs. FP, and FP vs. EP**.

Figure S1 **Rice callus**.

Figure S2 **Length distribution of CDS for unigenes predicted by BLAST and ESTScan**. Length distribution of nucleotide sequence **(A)** and protein sequence **(B)** predicted by BLAST and length distribution of nucleotide sequence **(C)** and protein sequence **(D)** predicted by ESTScan. Horizontal coordinates are sequence size and vertical coordinates are numbers of unigenes.

Video S1 **Three-dimensional scanning for cross-section of root tip, runnner hypha interweaved to form hyphal network**.

## Abbreviations

*P. liquidambari*: *Phomopsis liquidambari*
GFP: green fluorescent protein
Ck: pure culture/control treatment
EP: endophytic culture/ fungal mycelium of the *P. liquidambari* callus culture
FP: saprophytic culture/ litterfall-cultivated fungal mycelium of *P. liquidambari*
DEGs: differentially expressed genes
DGE: digital gene expression profiling
dai: days after inoculation
NR: non-redundant databases
KEGG: Kyoto Encyclopedia of Genes and Genomes
COG: Clusters of Orthologous Groups of proteins
GO: Gene Ontology
FDR: false discovery rate
CDS: Protein coding region.
