# Supplementary material for: De novo Transcriptome Assembly of Phomopsis liquidambari Provides Insights into Genes Associated with Different Lifestyles in Rice (Oryza sativa L.)
Source: Front Plant Sci. 2017 Feb 6;8:121. doi: 10.3389/fpls.2017.00121 (PMC5292412; doi:10.3389/fpls.2017.00121)
Supplement: Table S1 — qPCR primer sequences. [file Table1.PDF]

Table S1 qPCR primer sequences

Gene	Forward primer (5’ - 3’)	Reverse primer (5’ - 3’)
ITS (Bf1 and Br1)	CTGGCCCCCTCGGGGTCCCTGG	TTTCAGGGCCTGCCCTTTTACAGGC
β-actin	GACGACATGGAGAAGATCTGGCAC	CGTTGAACGTCTCGAAGACGATCTG
glutathione S-transferase Gst3	ACAAGAGCGGCGAGTTCAA	CGAGGACACGGTGATGAAGT
glutaminase a	GGATAGCCGCCACTTATACAC	CCTTGCCTTGAACTGGAGAC
NAD(P)-binding protein	GAACGACGCCAATCCATCAC	TACGAGAGGTTGCTGGTCAC
Probable lysosomal cobalamin transporter	CGAAGGACCAGCGTGAGAT	AAGACAGCCAGGAGCAAGAG
beta-glucosidase	GCTTCCTCCAGGCACAGAT	GATACCATTTCCGCACTCGTT
putative alcohol dehydrogenase	CCTTCGTGGATGGCAACAAT	TGTGGCAGTCTTCTCGTCAT
cytochrome P450 monooxygenase	TGGTGTGGTGGACGATTCTT	CTTGGTTGTGGCTAGACATCTC
major facilitator superfamily transporter	CGTTATCGTCTATGTCGTCCTC	TCCGTGTGCCGTTGTCTT
cyanide hydratase	GTCCGTCGAGGGTTTGAAGA	GTCAGCAATCACCTTGGTCAG
pyruvate decarboxylase	GTCTGTGGTGATGGTTCCTTC	GGCGTAGTTCCAGTTCTTGAT
heavy metal translocating P-type ATPase	ACGCAGAAGGCAGACAAGA	GACTTGACCAGCACGAACTC
transmembrane amino acid transporter	ATCGCCGCCGTCATCTATA	CCAGGAGGTCGTTGAACAC
glycoside hydrolase family 72 protein	TACTCCGCAGCAGATGTCTC	ACCGTAGCCAGTGAAGTTCTT
epl1 protein	CGGTGGATGAGCGAGACAT	GTGGTGGTGGTGATGATGATG
phosphoadenosine phosphosulfate reductase	CGAGATTGACGACGAGAG	CTTCCTACGACGAGACTTG
nucleolar protein nop-58	AGAAGACCGACGCATAGA	AACACGCCAGAAGGATTG
laccase-like protein	GGTCCATCGTCAACAACA	CGTATCGGTAGAGCATAGTC
high affinity copper transporter	GGCAGCAGTCATTCATCTA	GGAAGCACGAGTCTATTGT
3-ketoacyl-CoA reductase	GTCTATCTGGTCCTGTCCTA	ATTCCTTCACTCCTTCTTGG
glucan 1,4-alpha-maltohexaosidase	ATCAGCGACACCTATCAGA	GCCGTAAGACTCATCCATAA
