# Supplementary material for: De novo Transcriptome Assembly of Phomopsis liquidambari Provides Insights into Genes Associated with Different Lifestyles in Rice (Oryza sativa L.)
Source: Front Plant Sci. 2017 Feb 6;8:121. doi: 10.3389/fpls.2017.00121 (PMC5292412; doi:10.3389/fpls.2017.00121)
Supplement: Table S2 — Length distribution of contigs. [file Table2.PDF]

Table S2 Length distribution of contigs

sample	number/percent	100-200nt	200-300nt	300-400nt	400-500nt	>=500nt
P. liquidambari	number	25,877	7,261	4,147	2,255	11,580
	percent	50.62%	14.20%	8.11%	4.41%	22.65%
