# Supplementary material for: De novo Transcriptome Assembly of Phomopsis liquidambari Provides Insights into Genes Associated with Different Lifestyles in Rice (Oryza sativa L.)
Source: Front Plant Sci. 2017 Feb 6;8:121. doi: 10.3389/fpls.2017.00121 (PMC5292412; doi:10.3389/fpls.2017.00121)
Supplement: Table S3 — Length distribution of unigenes. [file Table3.PDF]

Table S3 Length distribution of unigenes

sample	number/ percent	100-500nt	500-1000nt	1000-1500nt	1500-2000nt	>=2000nt
P. liquidambari	number	15,072	7,138	3,942	2,275	3,997
	percent	46.48%	22.01%	12.16%	7.02%	12.33%
