# Supplementary material for: De novo Transcriptome Assembly of Phomopsis liquidambari Provides Insights into Genes Associated with Different Lifestyles in Rice (Oryza sativa L.)
Source: Front Plant Sci. 2017 Feb 6;8:121. doi: 10.3389/fpls.2017.00121 (PMC5292412; doi:10.3389/fpls.2017.00121)
Supplement: Table S4 — Functional annotation of unigenes. [file Table4.PDF]

Table S4 Function annotation of unigenes

Database	NR	NT	Swiss-Prot	KEGG	COG	GO	All
Annotated number	22,382	11,949	14,304	14,791	10,327	10,209	22,700
Annotated/total unigene	69.0%	36.9&	44.1%	45.6%	31.8%	31.5%	70.0%
