# Supplementary material for: De novo Transcriptome Assembly of Phomopsis liquidambari Provides Insights into Genes Associated with Different Lifestyles in Rice (Oryza sativa L.)
Source: Front Plant Sci. 2017 Feb 6;8:121. doi: 10.3389/fpls.2017.00121 (PMC5292412; doi:10.3389/fpls.2017.00121)
Supplement: Table S5 — GO classification of P. liquidambari unigenes. [file Table5.PDF]

Table S5 GO classification of *P. liquidambari* unigenes

Class	Number of *P. liquidambari*-Unigene
Biological process	
metabolic process	5532
cellular process	4554
localization	1546
establishment of localization	1539
biological regulation	626
regulation of biological process	604
response to stimulus	534
cellular component organization or biogenesis	343
single-organism process	301
signaling	279
multi-organism process	48
reproduction	29
developmental process	28
locomotion	28
negative regulation of biological process	24
reproductive process	24
positive regulation of biological process	10
multicellular organismal process	5
immune system process	5
death	5
carbon utilization	5
cell proliferation	4
growth	1
Cellular component	
cell	2458
cell part	2458
membrane	1486
organelle	1471
membrane part	989
macromolecular complex	747
organelle part	644
membrane-enclosed lumen	142
extracellular region	110
extracellular region part	12
nucleoid	5
extracellular matrix	2
virion	2
virion part	2
cell junction	1
Molecular function	
catalytic activity	6131

binding	5311
transporter activity	737
structural molecule activity	204
electron carrier activity	163
nucleic acid binding transcription factor activity	147
enzyme regulator activity	145
molecular transducer activity	119
receptor activity	48
antioxidant activity	47
nutrient reservoir activity	6
metallochaperone activity	4
