# Supplementary material for: De novo Transcriptome Assembly of Phomopsis liquidambari Provides Insights into Genes Associated with Different Lifestyles in Rice (Oryza sativa L.)
Source: Front Plant Sci. 2017 Feb 6;8:121. doi: 10.3389/fpls.2017.00121 (PMC5292412; doi:10.3389/fpls.2017.00121)
Supplement: Table S7 — Significantly enriched pathway in Ck vs. EP, Ck vs. FP, and FP vs. EP. [file Table7.PDF]

Table S7 Significantly enriched Pathway in Ck vs EP, Ck vs FP and FP vs EP

Pathway	Pathway ID	DEGs tested	P value	Q value
Ck vs FP				
Starch and sucrose metabolism	ko00500	212 (12.94%)	2.316605e-08	5.930509e-06
Legionellosis	ko05134	12 (0.73%)	0.0001248676	1.279288e-02
Type I diabetes mellitus	ko04940	4 (0.24%)	0.0001499166	1.279288e-02
Amino sugar and nucleotide sugar metabolism	ko00520	78 (4.76%)	0.002452099	1.569343e-01
African trypanosomiasis	ko05143	4 (0.24%)	0.01193849	5.018101e-01
Metabolic pathways	ko01100	537 (32.78%)	0.01278133	5.018101e-01
Ribosome	ko03010	52 (3.17%)	0.01592611	5.018101e-01
Butanoate metabolism	ko00650	31 (1.89%)	0.01633511	5.018101e-01
Porphyrin and chlorophyll metabolism	ko00860	14 (0.85%)	0.01764176	5.018101e-01
Two-component system	ko02020	38 (2.32%)	0.02130785	5.454810e-01
Prion diseases	ko05020	6 (0.37%)	0.02865271	6.668267e-01
Other types of O-glycan biosynthesis	ko00514	3 (0.18%)	0.04952802	9.172303e-01
Ck vs EP				
Butanoate metabolism	ko00650	32 (2.41%)	0.0003477344	0.06100926
Citrate cycle (TCA cycle)	ko00020	18 (1.35%)	0.0004675039	0.06100926
Synthesis and degradation of ketone bodies	ko00072	6 (0.45%)	0.001282817	0.11160508
Legionellosis	ko05134	9 (0.68%)	0.001976115	0.12560484
Parkinson's disease	ko05012	19 (1.43%)	0.002406223	0.1256048
Starch and sucrose metabolism	ko00500	149 (11.2%)	0.003393465	0.14761573
Ribosome	ko03010	46 (3.46%)	0.005423946	0.19025934
Oxidative phosphorylation	ko00190	28 (2.11%)	0.005831704	0.19025934
Prion diseases	ko05020	6 (0.45%)	0.01107491	0.32117239
Huntington's disease	ko05016	28 (2.11%)	0.02127829	0.52356956

Valine, leucine and isoleucine degradation	ko00280	15 (1.13%)	0.02206615	0.52356956
C5-Branched dibasic acid metabolism	ko00660	3 (0.23%)	0.04033481	0.79923476
Glucosinolate biosynthesis	ko00966	3 (0.23%)	0.04033481	0.79923476
Type I diabetes mellitus	ko04940	2 (0.15%)	0.04287083	0.79923476
FP vs EP				
Ribosome	ko03010	64 (4.26%)	1.990003e-06	0.0005174008
Ribosome biogenesis in eukaryotes	ko03008	36 (2.4%)	0.001307374	0.1699586200
Oxidative phosphorylation	ko00190	31 (2.06%)	0.005001116	0.4334300533
Protein processing in endoplasmic reticulum	ko04141	47 (3.13%)	0.0069754	0.4534010000
Citrate cycle (TCA cycle)	ko00020	16 (1.06%)	0.01090321	0.5028619143
Starch and sucrose metabolism	ko00500	161 (10.71%)	0.01266329	0.5028619143
Synthesis and degradation of ketone bodies	ko00072	5 (0.33%)	0.01353859	0.5028619143
Metabolic pathways	ko01100	489 (32.53%)	0.02827638	0.6039116353
Sesquiterpenoid and triterpenoid biosynthesis	ko00909	2 (0.13%)	0.02886406	0.6039116353
Valine, leucine and isoleucine degradation	ko00280	16 (1.06%)	0.02937736	0.6039116353
Carbon fixation pathways in prokaryotes	ko00720	5 (0.33%)	0.02990277	0.6039116353
Biosynthesis of secondary metabolites	ko01110	226 (15.04%)	0.03002212	0.6039116353
Sulfur metabolism	ko00920	10 (0.67%)	0.03047561	0.6039116353
GABAergic synapse	ko04727	7 (0.47%)	0.03284548	0.6039116353
Parkinson's disease	ko05012	17 (1.13%)	0.03555582	0.6039116353
Amino sugar and nucleotide sugar metabolism	ko00520	65 (4.32%)	0.03841584	0.6039116353
Two-component system	ko02020	34 (2.26%)	0.03948653	0.6039116353
Pantothenate and CoA biosynthesis	ko00770	12 (0.8%)	0.04840005	0.6991118333
