# Supplementary figures and images for: De novo Transcriptome Assembly of Phomopsis liquidambari Provides Insights into Genes Associated with Different Lifestyles in Rice (Oryza sativa L.)
Source: Front Plant Sci. 2017 Feb 6;8:121. doi: 10.3389/fpls.2017.00121 (PMC5292412; doi:10.3389/fpls.2017.00121)

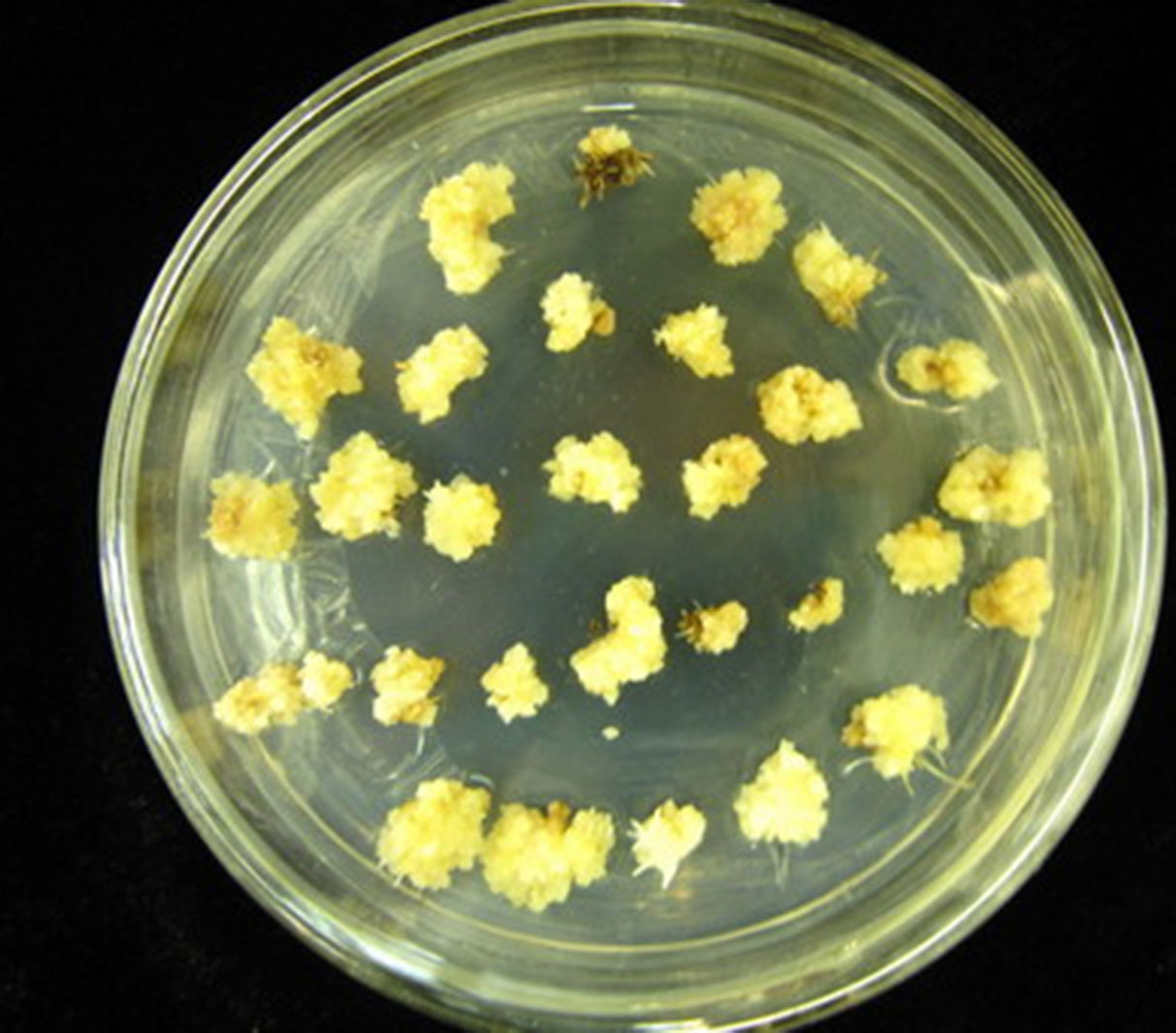

Supplement: Figure S1 — Rice callus. [file Image1.JPEG]

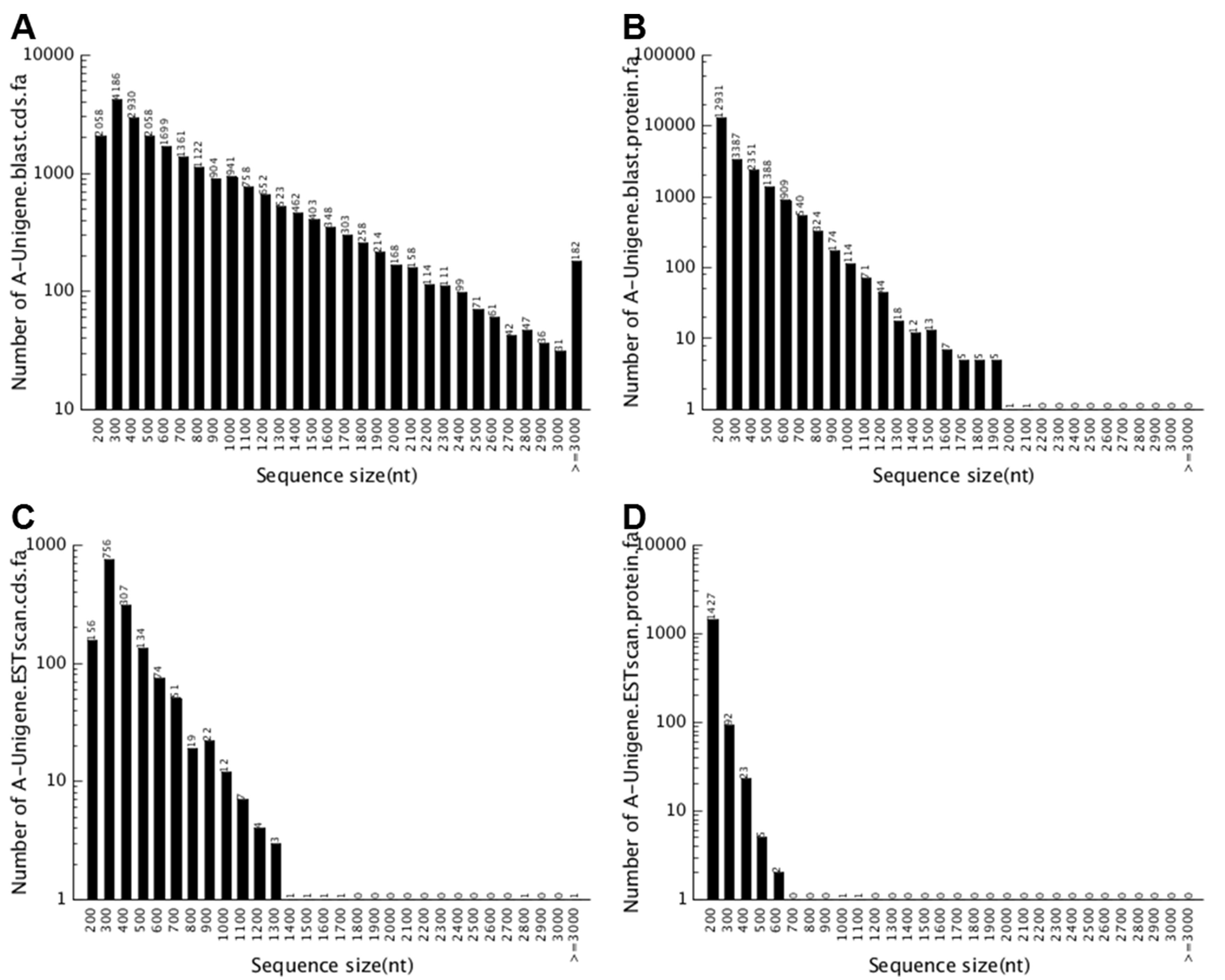

Supplement: Figure S2 — Length distribution of CDS for unigenes predicted by BLAST and ESTScan. Length distribution of nucleotide sequence (A) and protein sequence (B) predicted by BLAST and length distribution of nucleotide sequence (C) and protein sequence (D) predicted by ESTScan. Horizontal coordinates are sequence size and vertical coordinates are numbers of unigenes. [file Image2.JPEG]
